# Supplementary figures and images for: The superficial layers of the primary visual cortex create a saliency map that feeds forward to the parietal cortex
Source: PLoS Biol. 2025 Oct 14;23(10):e3003159. doi: 10.1371/journal.pbio.3003159 (PMC12543185; doi:10.1371/journal.pbio.3003159)

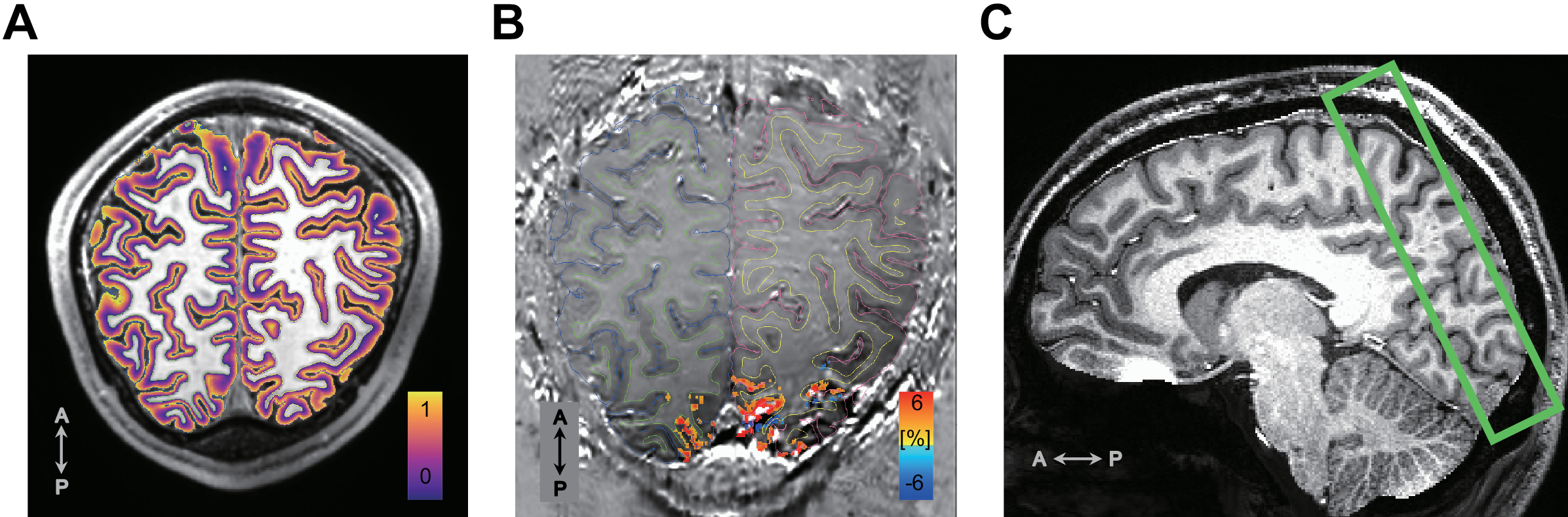

Supplement: S1 Fig — (A) Normalized cortical depth map overlayed on the T1w anatomical image in a representative participant. The equi-volume depth at 0 and 1 correspond to the WM and Pial surfaces, respectively. (B) VASO activations (90° + 15° + 0°, p < 0.001 uncorrected) overlayed on the mean VASO image. Green and yellow lines indicate the WM surface, while the blue and pink lines denote the pial surface. (C) The green box indicates the 3D slab of VASO fMRI acquisition. (TIF) [file pbio.3003159.s001.tif]

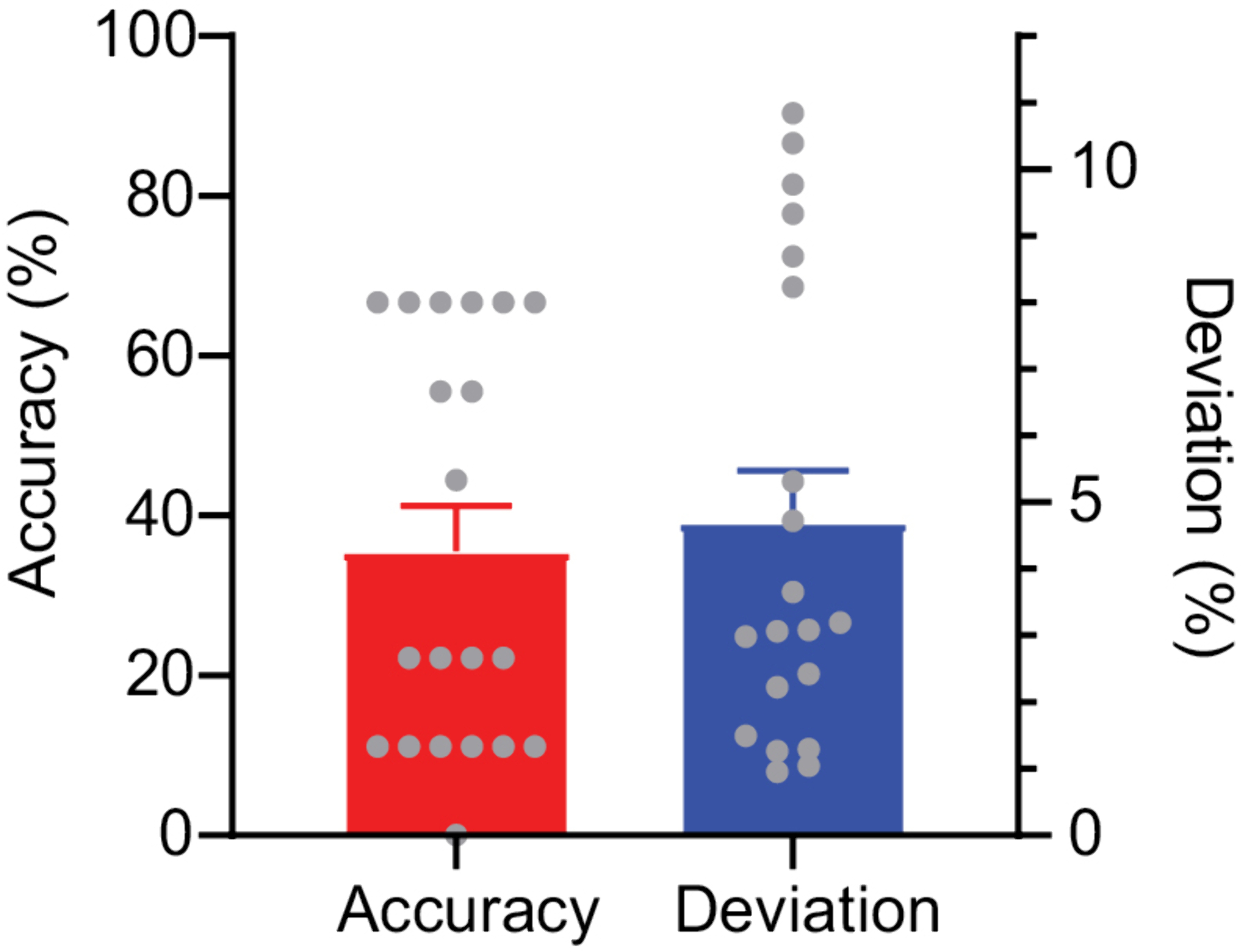

Supplement: S2 Fig — Accuracy represented the proportion of the runs that the participants gave the correct numbers of fixation changes. Deviation represented the difference between the giving numbers and the real numbers divided by the real numbers. The data underlying this Figure can be found in data/S2_data.xlsx at https://www.scidb.cn/doi/10.1101/2025.04.10.648136. (TIF) [file pbio.3003159.s002.tif]

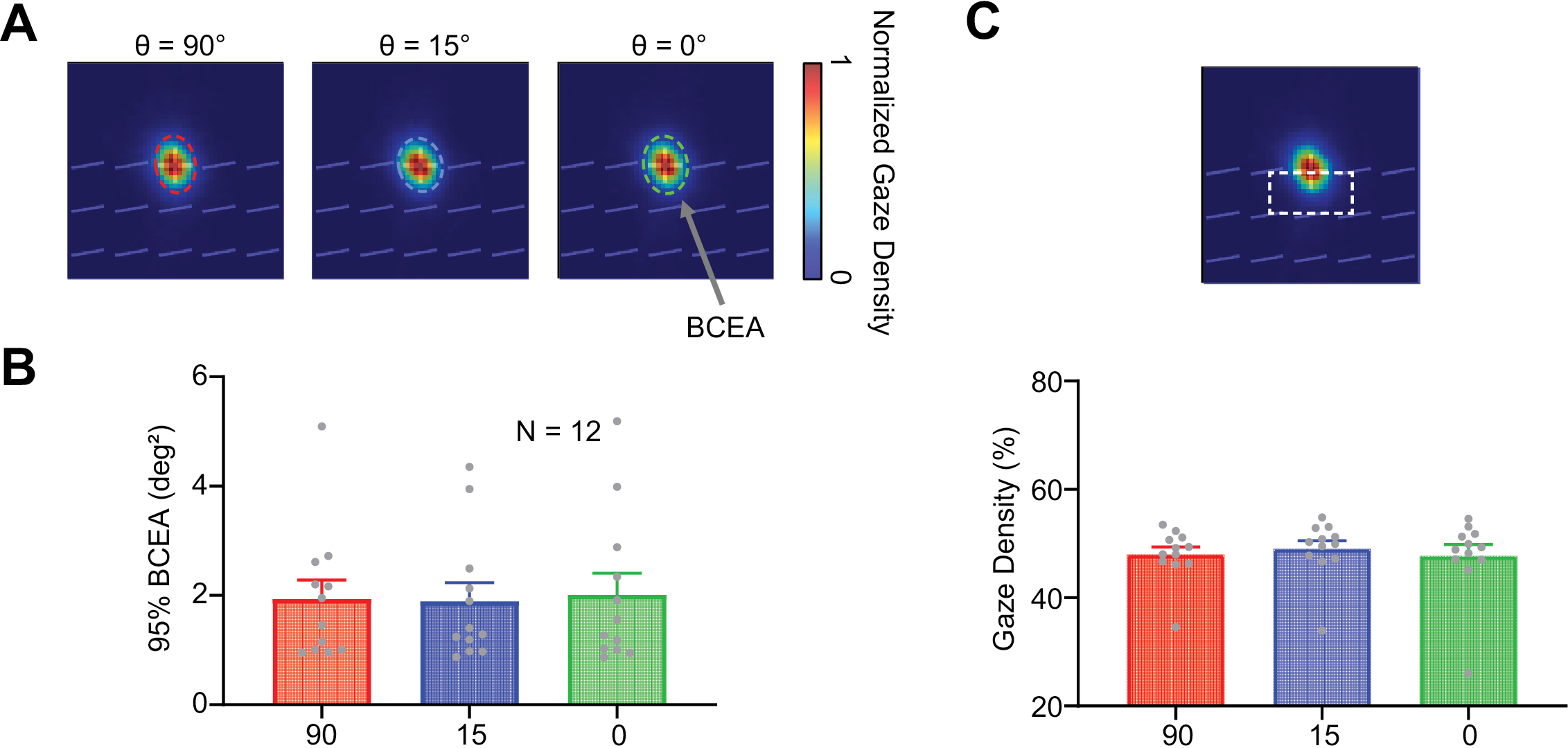

Supplement: S3 Fig — (A) The group-averaged heat maps of gaze density in the entire session. (B) The bivariate contour ellipse area (BCEA) of fixation distribution showed no significant difference across θ conditions (F2,22 = 0.291, p = 0.750, BF10 = 0.227, moderate evidence for H0: no difference across θ conditions). Error bars represent the standard deviation of the means. (C) The percentages of the gaze positions (gaze density) below the center of the fixation point showed no significant difference across θ conditions (F2,22 = 2.197, p = 0.135, BF10 = 0.736, weak evidence for H0). The dash lines indicate the region of the gaze positions selected for analysis. The data underlying this Figure can be found in data/S3_data.xlsx at https://www.scidb.cn/doi/10.1101/2025.04.10.648136. (TIF) [file pbio.3003159.s003.tif]

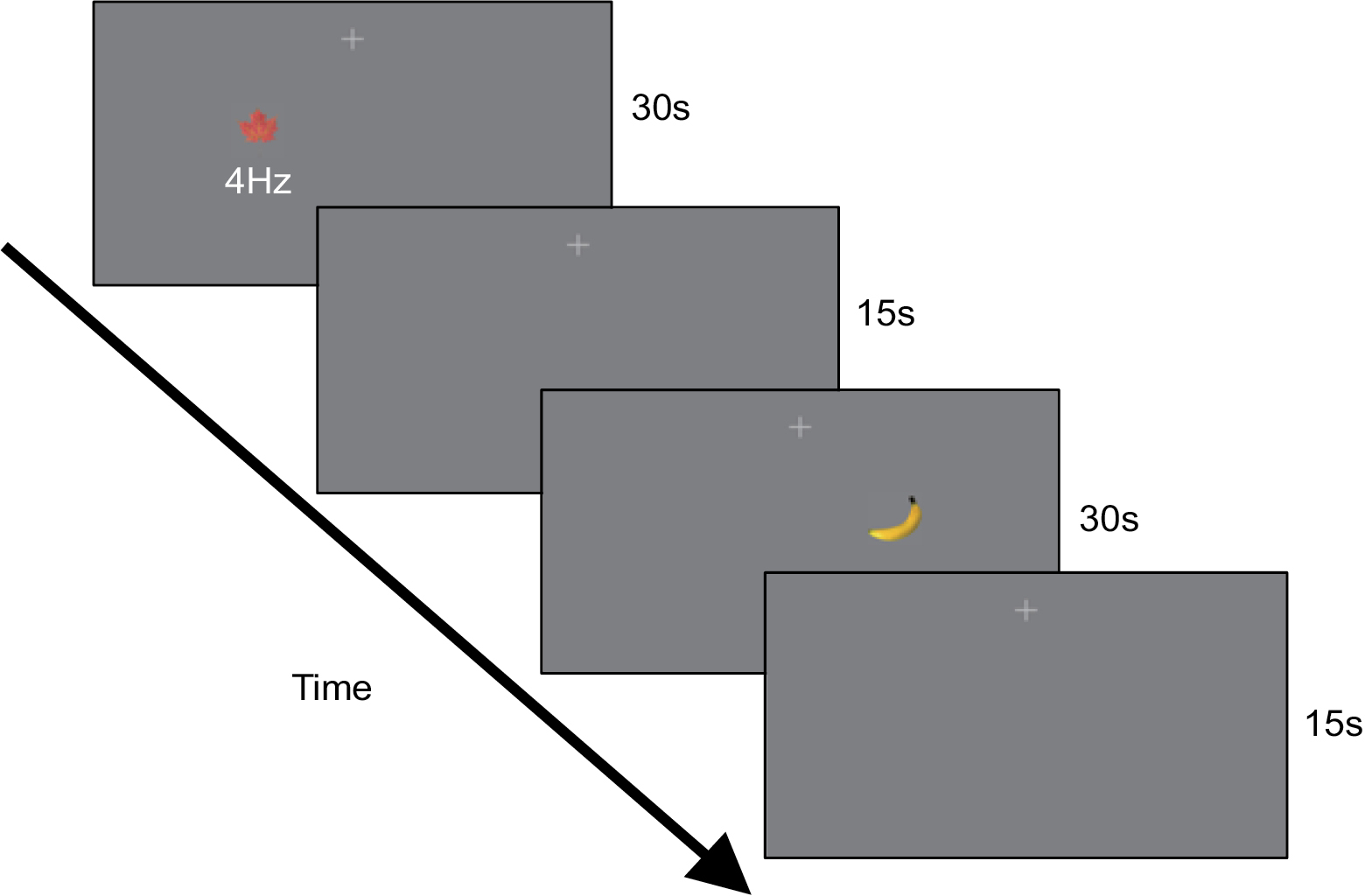

Supplement: S4 Fig — Naturalistic stimuli were presented at four images per second in the lower-left or lower-right quadrants in separate stimulus blocks, interleaved with 15-s fixation periods. The size and location of localizer stimuli matched the foreground region in the main experiment. (TIF) [file pbio.3003159.s004.tif]

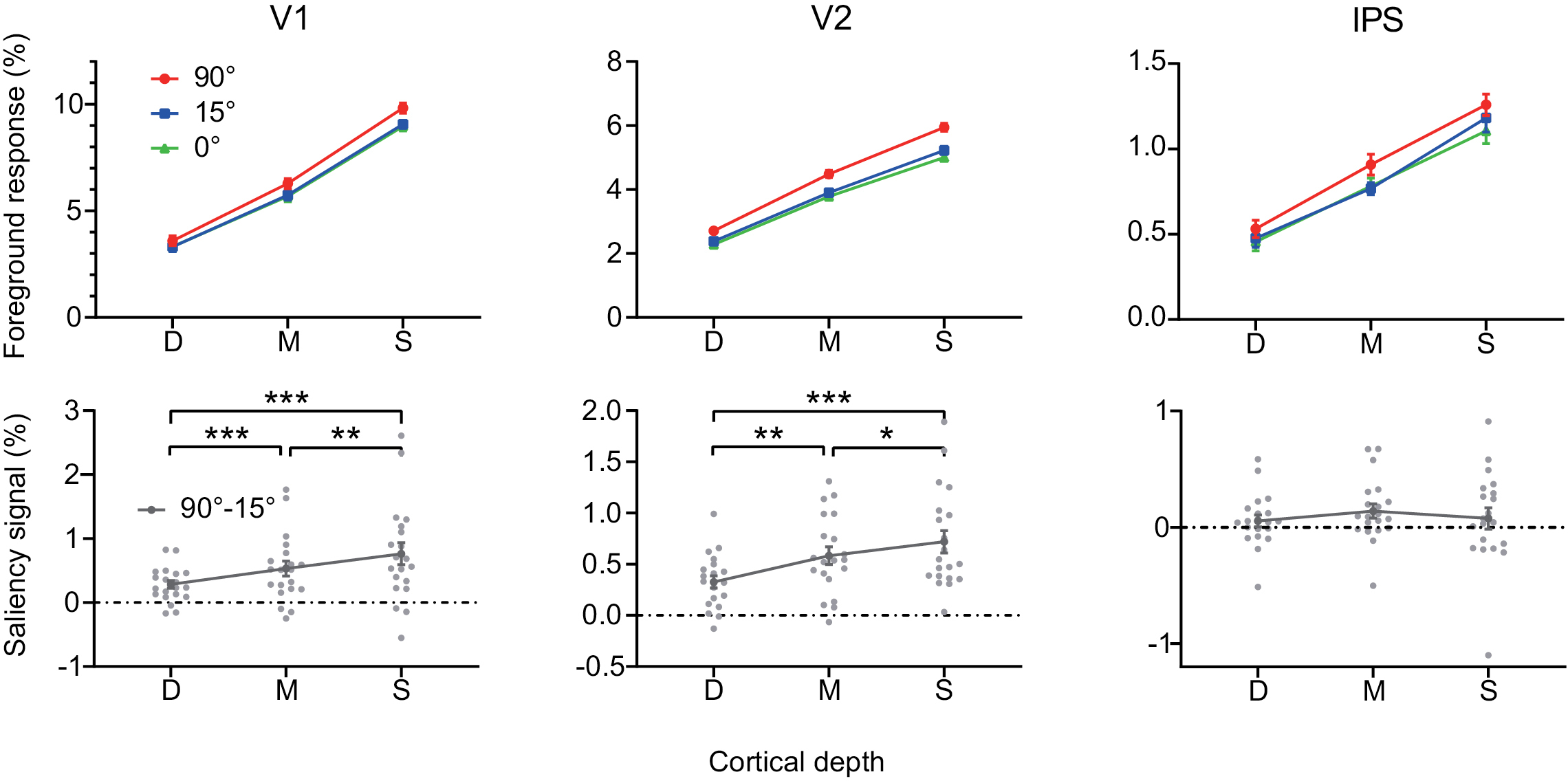

Supplement: S5 Fig — Top panel: BOLD response in different depths of V1, V2, and IPS in 90°, 15°, and 0° orientation contrast conditions; Bottom panel: Calculated from top panel, the response difference between 90° and 15° foregrounds. Error bars represent the standard deviation of the mean. *, ** and *** indicate p < 0.05, p < 0.01, p < 0.001. The data underlying this Figure can be found in data/S5_data.xlsx at https://www.scidb.cn/doi/10.1101/2025.04.10.648136. (TIF) [file pbio.3003159.s005.tif]

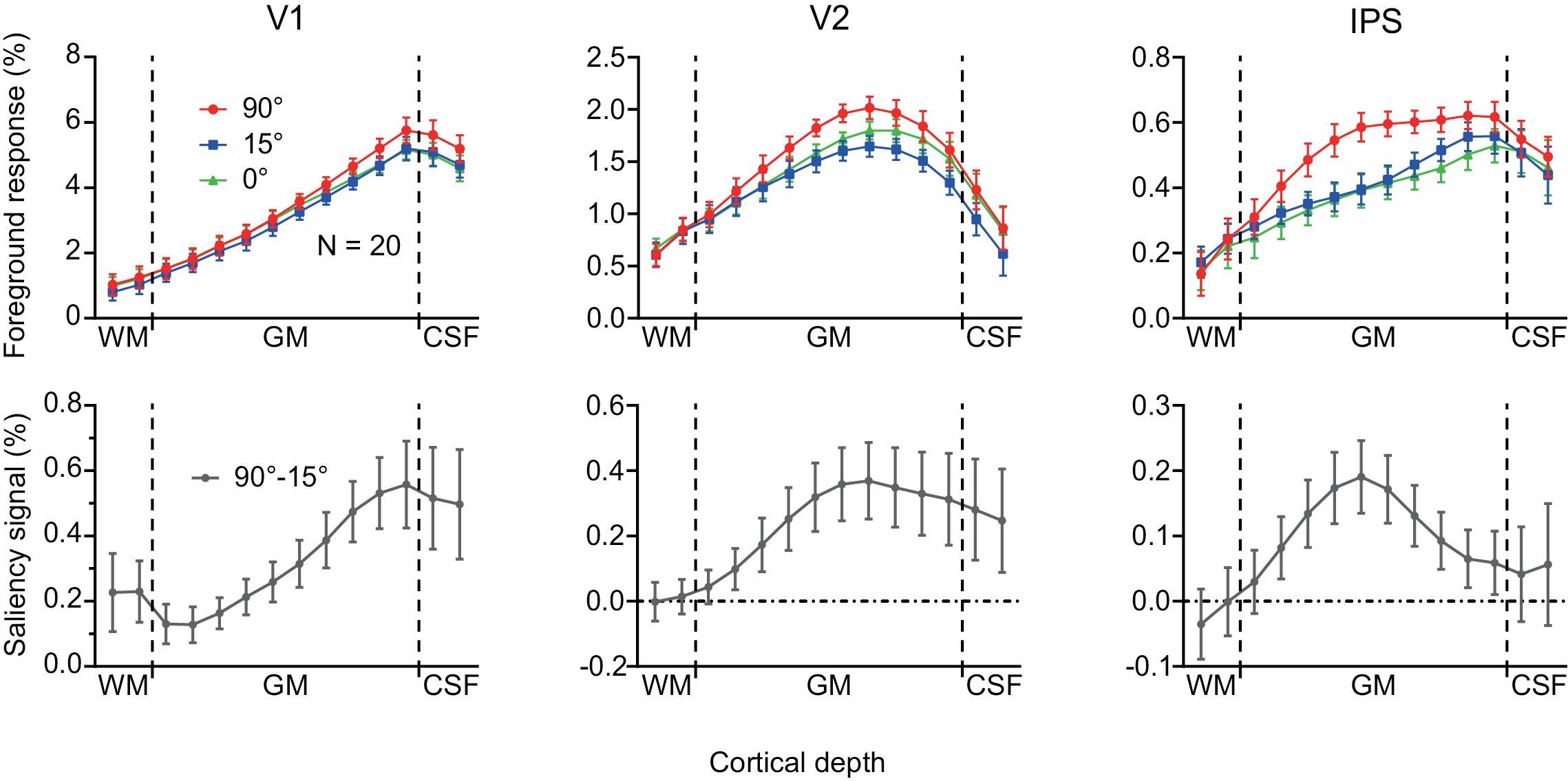

Supplement: S6 Fig — Top panel: CBV responses (SfMRI in percent signal change) to the orientation foregrounds in V1, V2, and IPS. Bottom panel: CBV response difference between the 90° and 15° foreground conditions (SSfMRI=SfMRI(90∘)−SfMRI(15∘)). Error bars represent SEM. WM: white matter, GM: gray matter, CSF: cerebrospinal fluid. (TIF) [file pbio.3003159.s006.tif]

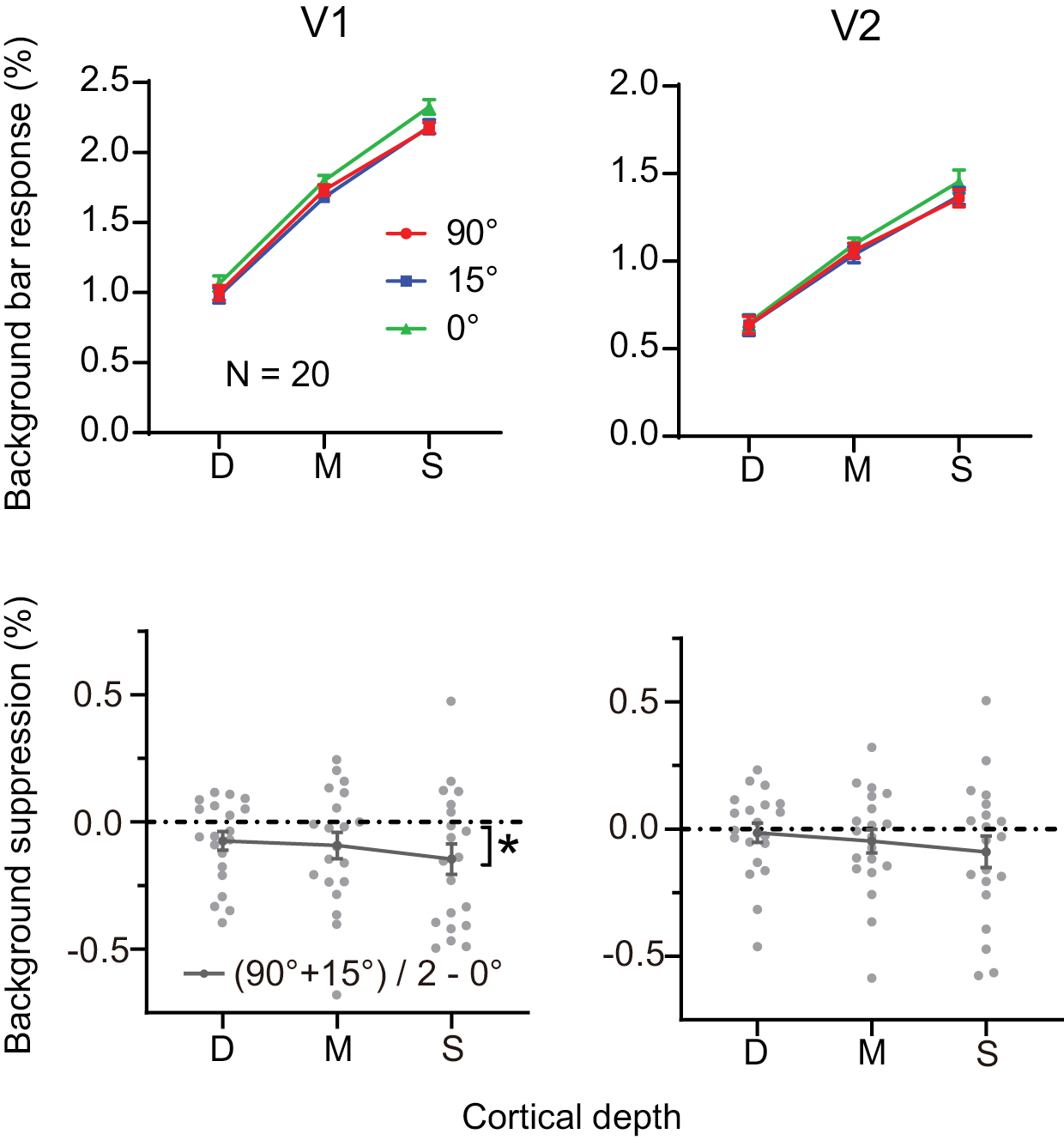

Supplement: S7 Fig — Upper: A significant effect of orientation contrast (θ = 90°, 15°, and 0°) was found in V1 (F2,38 = 3.700, p = 0.034) and a similar trend in V2, suggesting weaker background activity in the 90° and 15° conditions compared to the 0° or the uniform texture condition. No significant difference was found between the two θ conditions (F1,19 = 0.365, p = 0.553, BF10 = 5.601 × 10^−11^). Lower: The suppression effect was calculated as the response difference between the mean of 90° and 15° conditions and the 0° condition ((90° + 15°)/2 − 0°). A significant suppression effect was found only in the superficial depth of V1 (t19 = −2.877, p = 0.023, Holm corrected across cortical depths). These results suggest a suppression effect of background activity in the superficial layers of V1, independent with the orientation contrast between the foreground and the background bars. Each gray dot represents one participant. Error bars indicate SEM. * indicates p < 0.05. D, M, S indicate deep, middle, and superficial depth, respectively. The data underlying this Figure can be found in data/S7_data.xlsx at https://www.scidb.cn/doi/10.1101/2025.04.10.648136. (TIF) [file pbio.3003159.s007.tif]

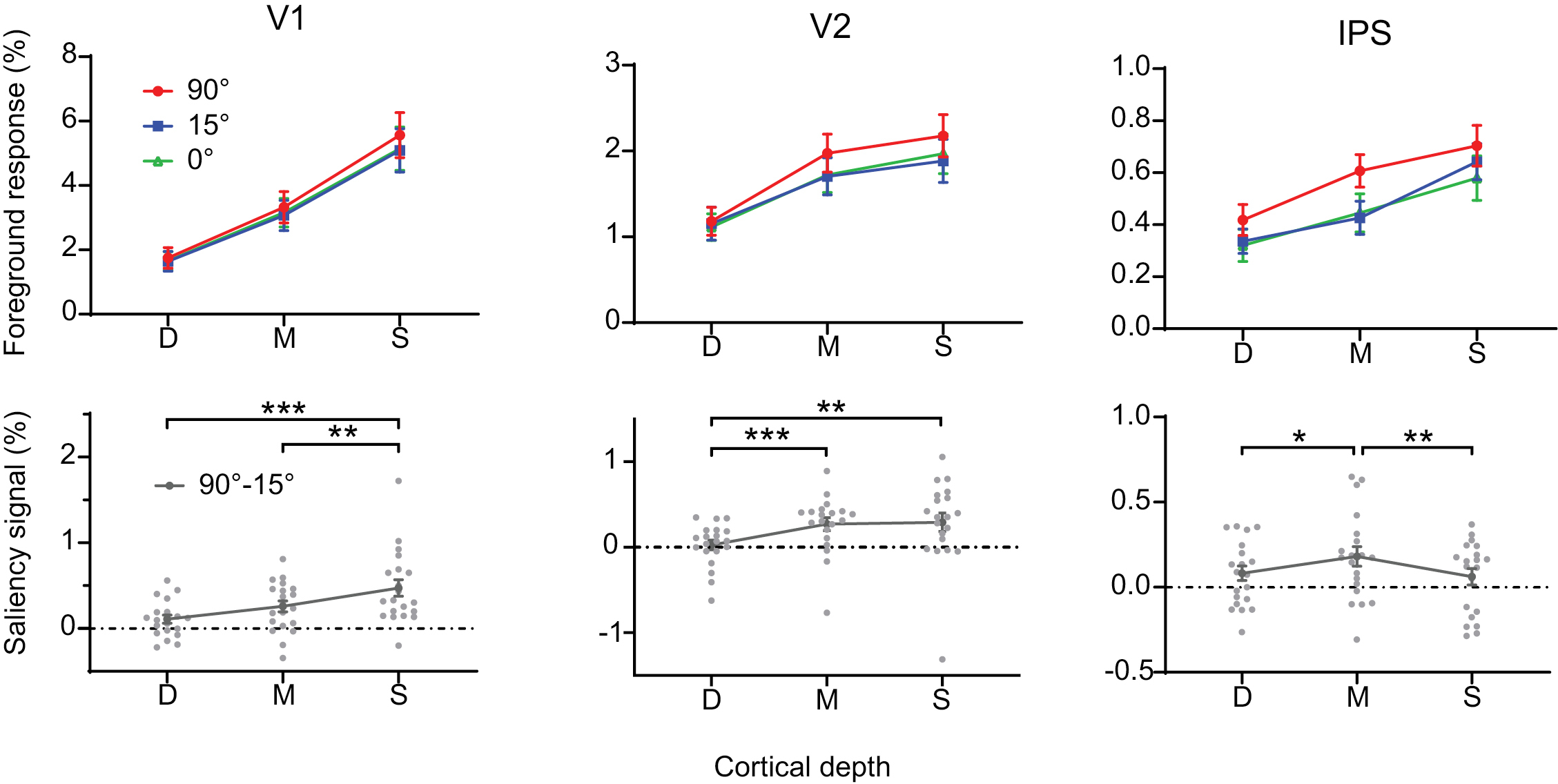

Supplement: S8 Fig — Conventions are identical as in Fig 3A. The data underlying this Figure can be found in data/S8_data.xlsx at https://www.scidb.cn/doi/10.1101/2025.04.10.648136. (TIF) [file pbio.3003159.s008.tif]

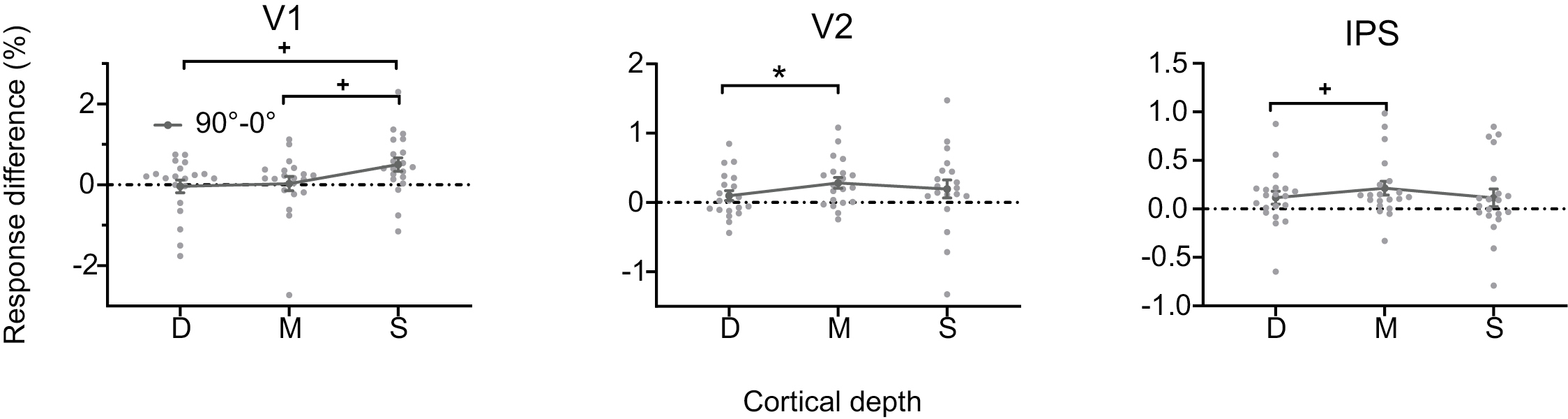

Supplement: S9 Fig — Similar laminar profile with Fig 3A bottom panel. Error bars represent the standard error of the mean. * p < 0.05, + p < 0.1. The data underlying this Figure can be found in data/S9_data.xlsx at https://www.scidb.cn/doi/10.1101/2025.04.10.648136. (TIF) [file pbio.3003159.s009.tif]

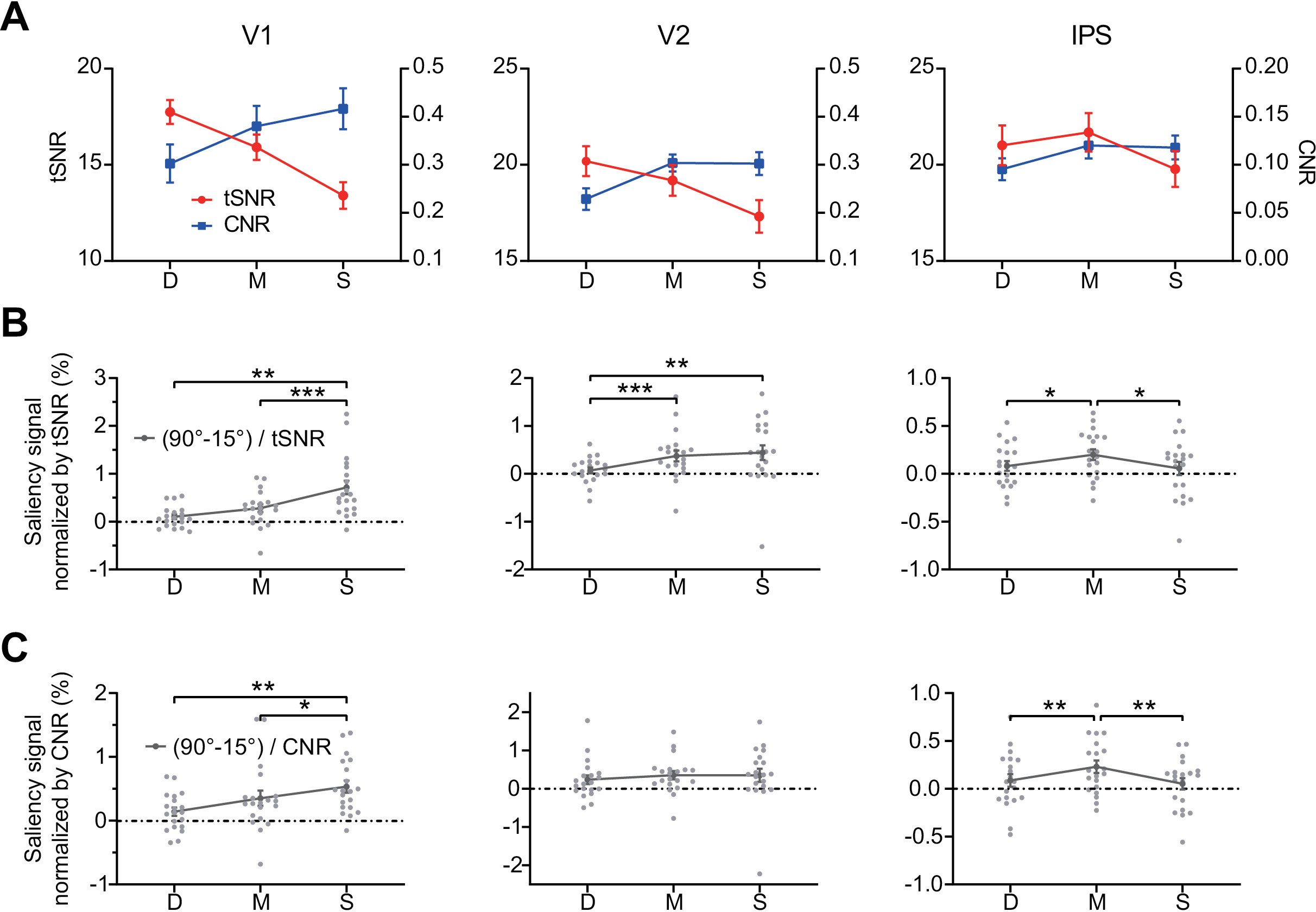

Supplement: S10 Fig — (A) The tSNR and CNR of CBV response across cortical depth in the foreground ROIs. tSNR: temporal signal-to-noise ratio, mean signal divided by the standard deviation. CNR: contrast-to-noise ratio, signal difference between stimulus and fixation periods divided by the standard deviation. (B) Saliency-sensitive responses normalized by tSNR. (C) Saliency-sensitive responses normalized by CNR. Error bars represent the standard error of the mean. *, **, and *** indicate p < 0.05, p < 0.01, and p < 0.001, respectively. The data underlying this Figure can be found in data/S10_data.xlsx at https://www.scidb.cn/doi/10.1101/2025.04.10.648136. (TIF) [file pbio.3003159.s010.tif]

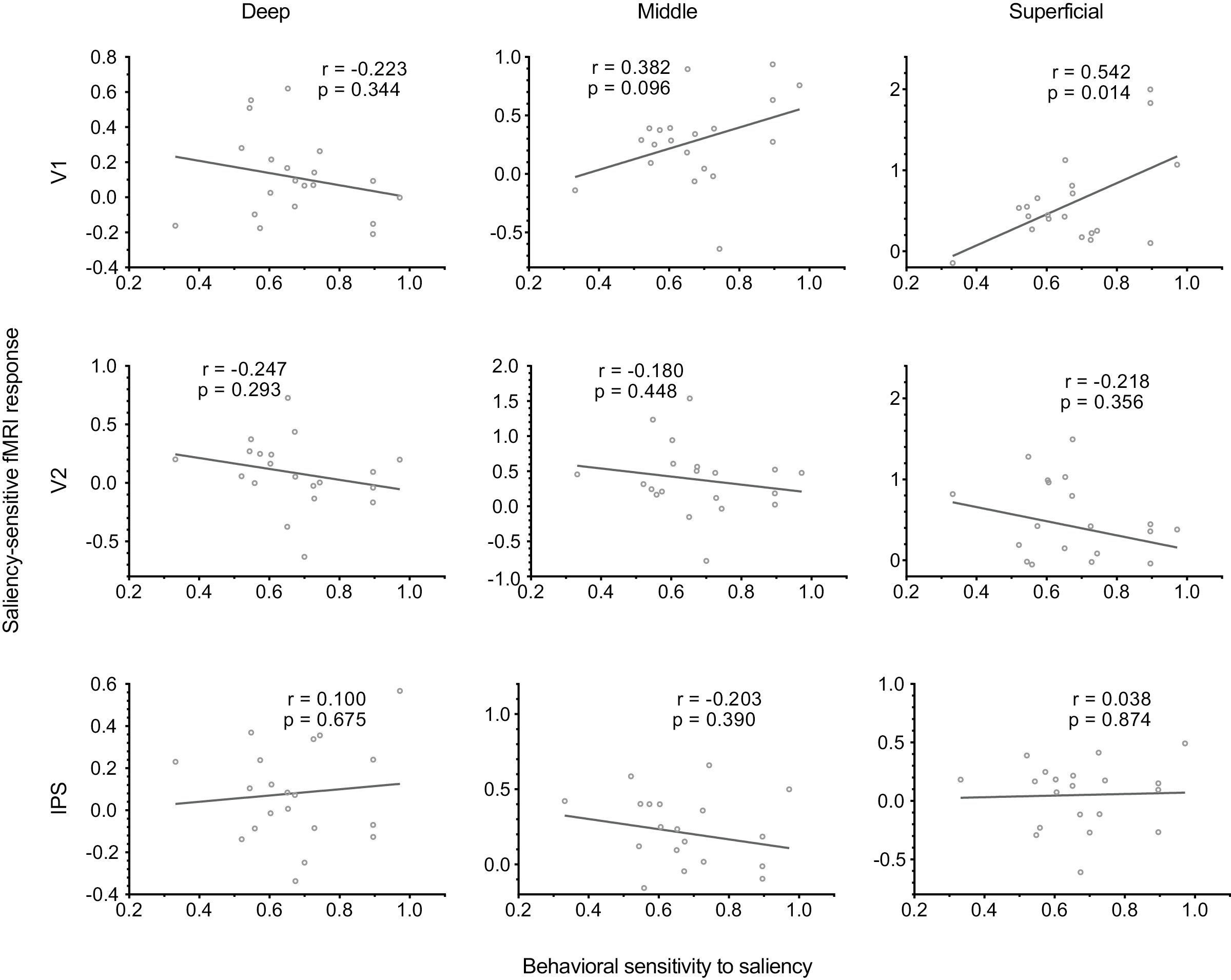

Supplement: S11 Fig — Each circle represents one participant. (TIF) [file pbio.3003159.s011.tif]

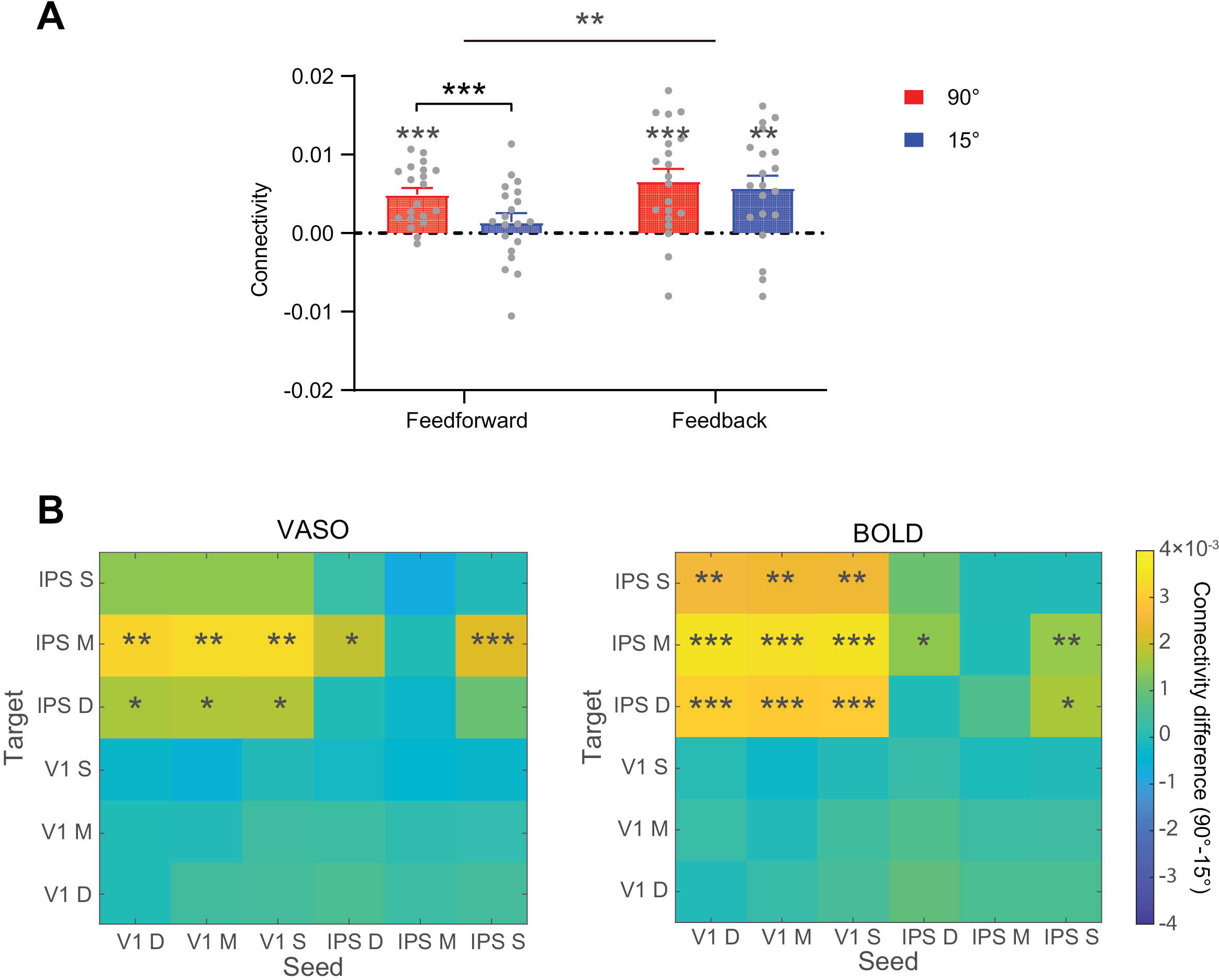

Supplement: S12 Fig — (A) Beta values of gPPI terms for the 90° and 15° conditions. Each gray dot represents one participant. Error bars represent the standard errors of the mean across participants. One sample t tests indicate significant positive connectivity in 90° of the feedforward pathways, 90° and 15° of the feedback pathways (feedforward 90°: t19 = 5.853, p < 0.001; feedforward 15°: t19 = 1.253, p = 0.225; feedback 90°: t19 = 4.350, p < 0.001; feedback 15°: t19 = 3.734, p = 0.001). Paired t tests indicate significant connectivity difference only in the feedforward pathway (feedforward 90° vs. 15°: t19 = 4.384, p < 0.001; feedback 90° vs. 15°: t19 = 2.041, p = 0.055). A two-way rm ANOVA shows a significant interaction between pathways (feedforward and feedback) and θ conditions (90° and 15°) (F1,19 = 10.067, p = 0.005). (B) The gPPI connectivity matrix across three cortical depths in V1 and IPS. Columns and rows correspond to the seed and target ROIs, respectively. The color scale indicates the beta difference of interaction terms (beta(90°) − beta(15°)). * p < 0.05, ** p < 0.01, *** p < 0.001, uncorrected. The data underlying this Figure can be found in data/S12_data.xlsx at https://www.scidb.cn/doi/10.1101/2025.04.10.648136. (TIF) [file pbio.3003159.s012.tif]

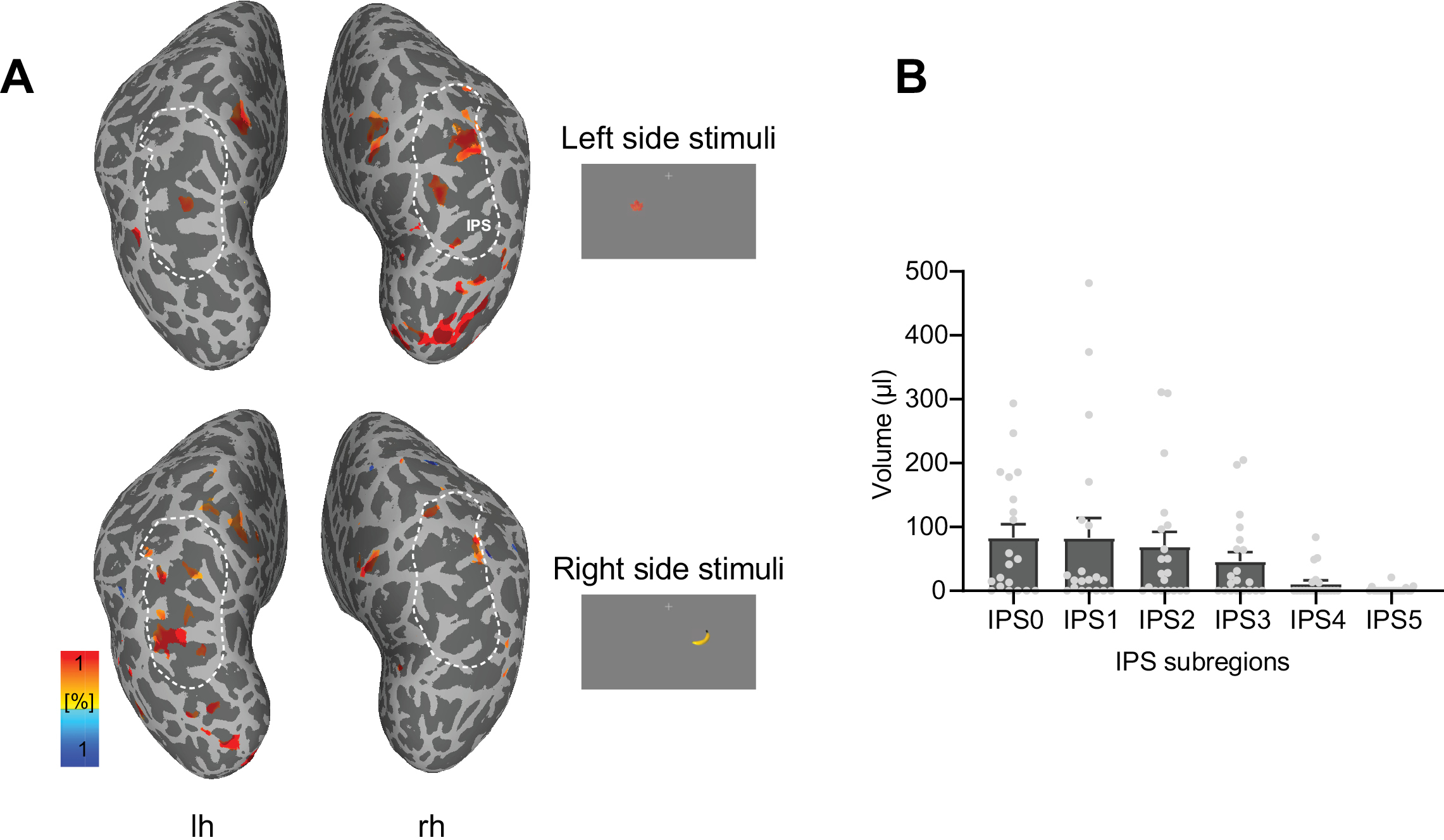

Supplement: S13 Fig — (A) Localizer activations to the left- or right-side stimuli in the IPS of a representative participant. Dashed lines mark the borders of the IPS. (B) Group-averaged ROI volume in IPS subregions. Error bars represent SEM across participants. Each gray dot represents data from one participant. The data underlying this Figure can be found in data/S13_data.xlsx at https://www.scidb.cn/doi/10.1101/2025.04.10.648136. (A) Localizer activations to the left- or right-side stimuli in the IPS of a representative participant. Dashed lines mark the borders of the IPS. (B) Group-averaged ROI volume in IPS subregions. Error bars represent SEM across participants. Each gray dot represents data from one participant. The data underlying this Figure can be found in data/S13_data.xlsx at https://www.scidb.cn/doi/10.1101/2025.04.10.648136. (TIF) [file pbio.3003159.s013.tif]

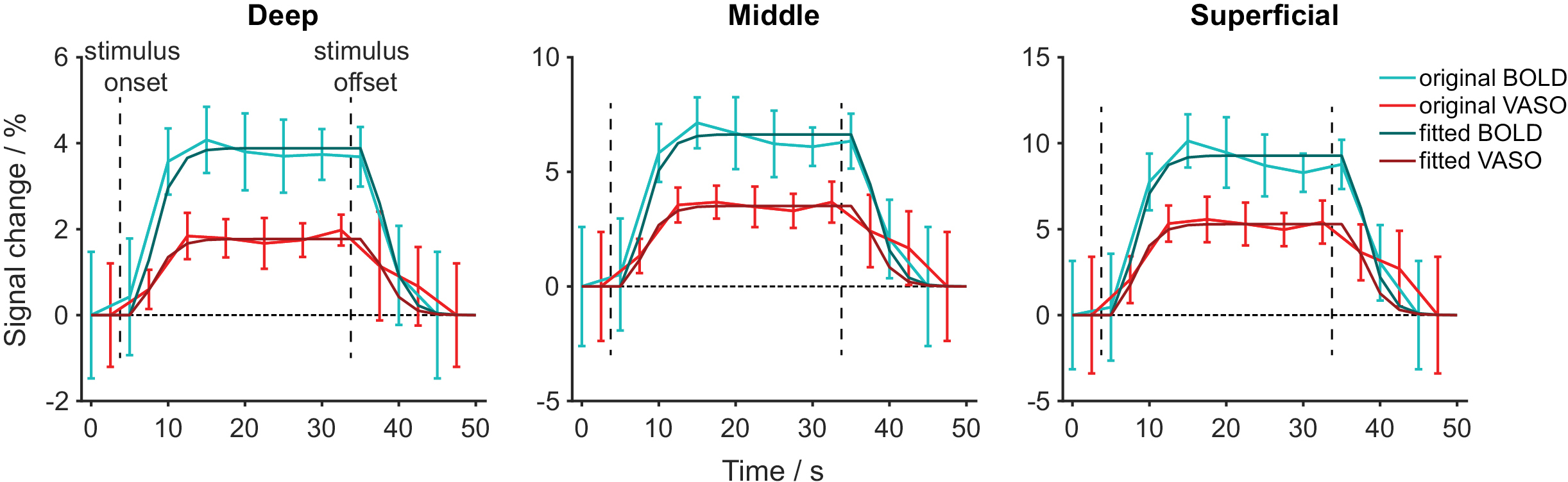

Supplement: S14 Fig — Error bars represent SEM across participants. (TIF) [file pbio.3003159.s014.tif]
