# Supplementary material for: The superficial layers of the primary visual cortex create a saliency map that feeds forward to the parietal cortex
Source: PLoS Biol. 2025 Oct 14;23(10):e3003159. doi: 10.1371/journal.pbio.3003159 (PMC12543185; doi:10.1371/journal.pbio.3003159)
Supplement: Attachment — Submitted filename: Response to reviewers.docx [file pbio.3003159.s017.docx]

Reviewer #1: This study uses laminar fMRI to investigate whether saliency signals are generated in V1, Superior Colliculus or in IPS, as a way to arbitrate between competing hypotheses in the literature. Different laminar profiles of saliency modulation would be expected depending on the source of the saliency signal. High-field fMRI in combination with a CBV-weighted VASO technique is employed to measure activation in different cortical layers while participants are presented with low-level stimuli varying in saliency. The results show that saliency effects were strongest in superficial layers in V1 and in middle layers in IPS, in line with the V1 Saliency Hypothesis proposing that saliency is generated in V1. 

The paper is well written and uses state-of-the-art methods to investigate a clearly defined and interesting question. I was especially impressed by the clarity with which the hypotheses were formulated and investigated, although I also have some questions about that (see below). That being said, I still have a few concerns that I would like to see addressed. 

Major comments:
The hypotheses entirely rely on the assumption that the involvement of specific layers corresponds to the direction of communication (forward or backward). The authors cite the classic Felleman and van Essen (1991) study to motivate their laminar hypotheses (line 116). However, is this the only work supporting the direction of communication between the layers of different cortical cites or has other research provided convergent findings? For example, how many studies have shown that feedback connections from parietal to V1 go from deep to deep and superficial layers? To be able to interpret the results, it is crucial to indicate how solid the evidence for this assumption is.  
Re: Thank you for highlighting the need to provide stronger evidence supporting the assumed relationship between laminar involvement and the direction of cortico-cortical communication. In addition to the meta-analysis by Felleman and van Essen (1991), more recent studies have also provided converging anatomical and functional evidence for the hierarchical organization of cerebral cortex. For example, Shipp (2007) and Markov et al. (2014) report that feedforward connections typically originate from superficial layers and terminate in middle layers, while feedback connections arise from deep layers and project to both deep and superficial layers. Specifically, many studies have found this principle in occipital visual cortex and posterior temporal cortex (e.g. Anderson & Martin, 2009; Federer et al., 2021; Rockland & Pandya, 1979; Rockland & Virga, 1989), and Markov et al. extended it to frontal and parietal cortex (FEF, DP, and LIP et al.) (Markov et al., 2013, 2014).

We have now cited the representative studies in the revised manuscript (lines 118–122) to strengthen the evidence for our hypotheses: *"* *Figure 1 schematizes the laminar patterns of saliency-related signals in V1 and parietal cortex according to each of the three possible origins of the saliency signal: parietal cortex, V1, and SC, based on the canonical neural circuits in the primate visual system (Felleman & Van Essen, 1991; Markov, Vezoli, et al., 2014; Rockland & Pandya, 1979; Shipp, 2007)".*

Reference:

Anderson, J. C., & Martin, K. A. C. (2009). The Synaptic Connections between Cortical Areas V1 and V2 in Macaque Monkey. *The Journal of Neuroscience*, 29(36), 11283–11293. https://doi.org/10.1523/jneurosci.5757-08.2009

Federer, F., Ta’afua, S., Merlin, S., Hassanpour, M. S., & Angelucci, A. (2021). Stream-specific feedback inputs to the primate primary visual cortex. *Nature Communications*, 12(1). https://doi.org/10.1038/s41467-020-20505-5

Felleman, D. J., & Van Essen, D. C. (1991). Distributed Hierarchical Processing in the Primate Cerebral Cortex. *Cerebral Cortex*, 1(1), 1–47. https://doi.org/10.1093/cercor/1.1.1

Markov, N. T., Ercsey-Ravasz, M., Van Essen, D. C., Knoblauch, K., Toroczkai, Z., & Kennedy, H. (2013). Cortical High-Density Counterstream Architectures. *Science*, 342(6158), 1238406. https://doi.org/10.1126/science.1238406

Markov, N. T., Vezoli, J., Chameau, P., Falchier, A., Quilodran, R., Huissoud, C., Lamy, C., Misery, P., Giroud, P., Ullman, S., Barone, P., Dehay, C., Knoblauch, K., & Kennedy, H. (2014). Anatomy of hierarchy: Feedforward and feedback pathways in macaque visual cortex. *Journal of Comparative Neurology*, 522(1), 225–259. https://doi.org/10.1002/cne.23458

Rockland, K. S., & Pandya, D. N. (1979). Laminar origins and terminations of cortical connections of the occipital lobe in the rhesus monkey. *Brain Research*, 179(1), 3–20. https://doi.org/10.1016/0006-8993(79)90485-2

Rockland, K. S., & Virga, A. (1989). Terminal arbors of individual “Feedback” axons projecting from area V2 to V1 in the macaque monkey: A study using immunohistochemistry of anterogradely transported Phaseolus vulgaris‐leucoagglutinin. *Journal of Comparative Neurology*, 285(1), 54–72. https://doi.org/10.1002/cne.902850106

Shipp, S. (2007). Structure and function of the cerebral cortex. *Current Biology*, 17(12), R443–R449. https://doi.org/10.1016/j.cub.2007.03.044

Relatedly, it was unclear to me whether the hypothesized laminar profiles in Fig. 1 were generated using some kind of computational model or illustrated by hand based on the descriptions in the literature? Please clarify in the text. 
Re: Thank you for raising this important point. The hypothesized laminar profiles in Figure 1 were manually illustrated based on the hierarchical connections across brain regions. We have added the corresponding description to the legend of Figure 1 (lines 115-116) in the revised manuscript: *"These indicated relative strengths are schematic representations derived from existing literature on the hierarchical connections of brain regions.".*

I was a bit surprised to read that the analyses focused on the laminar profiles in V1, V2 and IPS while the introduction and hypotheses are focused on V1, SC and IPS. Eventually I realized that SC was not included because the effects of SC being the source would be reflected in the laminar profile of V1. However, it was still not entirely clear to me why V2 was included. It would be helpful if the authors could emphasize the relationship between SC being the source and the laminar profile in V1 and also further explain the relationship between the hypotheses and the laminar profile in V2.
Re: Thank you for bringing this important issue to our attention. In our study, SC activity was not recorded due to the small field-of-view of the VASO fMRI technique. However, as you noted, the laminar profile of V1 can help distinguish between the hypotheses that either V1 or SC is the primary source of saliency signals. According to the SC hypothesis, saliency-related activity would be stronger in the middle layer. We have added an explanation to clarify and emphasize this relationship in the Introduction (lines 132–137): *"Specifically, this pathway may relay through the pulvinar, terminating in the middle and superficial cortical layers, or through the LGN, which primarily targets the middle layers of V1. Overall, the middle layer receives stronger thalamic input than the superficial layer (Shipp, 2003). Thus, although the SC was not recorded in the current study due to the limited field-of-view of VASO fMRI, the laminar profiles of saliency signals recorded in the cortical regions can still be used to test the hypothesis whether these signals originate from a subcortical source."*.

Area V2 serves as an intermediate stage in the transmission of saliency signals between V1 and IPS. It receives input from V1 primarily in its middle layers and sends output from its superficial layers to higher cortex, including IPS. Therefore, the laminar pattern of saliency in V2 is expected to lie between that of V1 and IPS. It could provide further support evaluating the hypotheses of saliency origin and helps determine whether the laminar profiles observed in V1 and IPS are unique to these areas. We have added this explanation before presenting the layer-fMRI results (lines 206–209):*"As an intermediate level in the cortical hierarchy between V1 and IPS, V2 was expected to exhibit a laminar profile in-between those of the two brain regions. Thus, the laminar pattern of V2 can provide further information to distinguish the laminar hypotheses schematized in Figure 1."*

I found it surprising that the saliency effect in V1, but not IPS, predicted the behaviour since we might a priori expect that responses in more downstream regions, closer to the output, are more predictive. Could the participants explain how this might work? Relatedly, between-subject correlations with such a small N are notoriously underpowered (e.g. see Marek et al., 2022 Neuron), which should be mentioned.
Re: We thank the reviewer for the insightful comment. The finding that the saliency effect in V1, but not IPS, predicted behavior may reflect task differences between the behavioral and fMRI experiments. In the behavioral experiment, participants responded directly to the foreground bars, whereas in the fMRI experiment, they performed a central fixation task in which the foreground bars were task irrelevant. Since IPS activity strongly depends on task demand, the fMRI activity during the fixation task can be less predictive for the task performance in the behavioral experiment. In contrast, V1 activity is much less task-sensitive and thus can better predict the salience effect in the behavioral experiment.

We agree that between-subject correlations with small sample sizes are statistically underpowered, as highlighted by Marek et al. (2022). However, our analyses were hypothesis-driven and limited to predefined regions of interest (V1 and IPS), rather than exploratory whole-brain correlation analysis. This targeted approach reduces the risk of false positives and does not require the same sample size as large-scale connectome studies. Moreover, we applied non-parametric permutation tests to control for multiple comparisons. These points have been added to the revised manuscript (lines 299–302): *"Although the sample size (N = 20) of this study is not sufficient for an exploratory whole-brain correlation analysis with behavioral performance, as highlighted by (Marek et al., 2022), it provides enough power for hypothesis-driven analyses focused on a limited number of brain regions (Zhang et al., 2012)."*

Reference:

Marek, S., Tervo-Clemmens, B., Calabro, F. J., Montez, D. F., Kay, B. P., Hatoum, A. S., Donohue, M. R., Foran, W., Miller, R. L., Hendrickson, T. J., Malone, S. M., Kandala, S., Feczko, E., Miranda-Dominguez, O., Graham, A. M., Earl, E. A., Perrone, A. J., Cordova, M., Doyle, O., … Dosenbach, N. U. F. (2022). Reproducible brain-wide association studies require thousands of individuals. *Nature*, 603(7902), 654–660. https://doi.org/10.1038/s41586-022-04492-9

Zhang, X., Zhaoping, L., Zhou, T., & Fang, F. (2012). Neural Activities in V1 Create a Bottom-Up Saliency Map. *Neuron*, 73(1), 183–192. https://doi.org/10.1016/j.neuron.2011.10.035

I was confused by the PPI analysis. The methods state that the saliency-dependent connectivity was defined as the difference between the PPI beta weights of the two conditions. However, shouldn't the PPI weight already be defined as an interaction between the time series and the difference between the two conditions (<https://pubmed.ncbi.nlm.nih.gov/9344826/>)? In that case, the PPI beta itself would already show the condition effect. Furthermore, in standard PPI, the analysis is executed whole-brain. How exactly is an ROI target analysis implemented here? 
Re: Thank you for bringing this issue to our attention. As you mentioned, in the standard PPI approach, the PPI beta already reflects the condition effect because the interaction term is computed using a contrast vector (where one condition is coded as 1 and the other as –1). However, more recent work has introduced an extended approach known as generalized PPI (gPPI), which estimates separate interaction beta weights for each condition. Although the interaction estimates in gPPI are conceptually comparable to those in standard PPI, the gPPI framework provides improved model fit and accommodates models with more than two conditions (McLaren et al., 2012). Figure R1 offers a helpful illustration of the differences between these two methods.

In our analysis, the difference between the gPPI beta weights for the two conditions (i.e., the beta contrast) is equivalent to the condition effect beta in the standard PPI approach. PPI can be used to assess how brain regions interact in a task-dependent manner, and here we used it to characterize how the connectivity between V1 and IPS was modulated by saliency.

**Figure R1.** A comparison of two PPI methods for detecting context-modulated functional connectivity in fMRI. The sPPI provides a single interaction term describing the extent to which task conditions affect the functional connectivity between two brain regions. The gPPI is similar to the sPPI, except that instead of contrasting the stimulus conditions in a single beta estimate, the interaction terms for different stimulus conditions are estimated separately and the contrast of betas was calculated as the gPPI. (Adapted from Cisler et al., 2014)

Reference:

McLaren, D. G., Ries, M. L., Xu, G., & Johnson, S. C. (2012). A generalized form of context-dependent psychophysiological interactions (gPPI): A comparison to standard approaches. *NeuroImage*, *61*(4), 1277–1286. https://doi.org/10.1016/j.neuroimage.2012.03.068

Cisler, J. M., Bush, K., & Steele, J. S. (2014). A comparison of statistical methods for detecting context-modulated functional connectivity in fMRI. *NeuroImage*, *84*, 1042–1052. https://doi.org/10.1016/j.neuroimage.2013.09.018

Minor comments: 
For the fMRI task, was the participant adjusted luminance used or just black and white stimuli? 
Re: In the fMRI experiment, the stimuli and background were presented in black and white to maximize the SNR of fMRI signals to the visual stimuli.

We have added this description in presenting the revised Method section (lines 495–499):*"* *Visual stimuli were identical as those in the psychophysical experiment except that the luminance of the bars and the uniform background were*$L_{\mathrm{bar}}$ *= 87.5 cd/m^2^ and*$L_{0}$ *= 0.4 cd/m^2^ (max and min luminance of the projector, a high contrast texture stimulus was used to maximize the SNR of fMRI signals to the visual stimuli), respectively."*

Line 297 stating that the neural source of saliency has been controversial should be followed with references to this debate. 
Re: Thank you for pointing this out. We have added references to relevant studies discussing this debate, which are now cited in lines 368–369.

Motivations for the study are discussed in the discussion (starting line 319) that would be better placed in the introduction.  
Re: Thank you for this helpful suggestion. We agree that limitations of previous studies were indeed more appropriate presented as a part of the study's motivation. We have moved these to the Introduction (lines 72–84).


Reviewer #2: This study leverages 7T laminar fMRI to investigate the neural substrates of visual saliency, revealing robust saliency-related signals in superficial layers of V1 and middle layers of IPS, alongside feedforward information flow from V1 to IPS. Further, the saliency-related activity in superficial V1 layers can predict participants' behavioral performance. Overall, the research question is interesting and the manuscript is well-written. Several points require clarification to strengthen the interpretation and technical rigor.

1 the introduction posits three potential hypotheses (V1, IPS, SC) for saliency computation. While substantial empirical support is provided for the first two hypotheses, the rationale for the third hypothesis involving subcortical pathways is not very clear. Given the established role of the superior colliculus (SC) and pulvinar in attentional processes, it would be valuable to clarify whether the term "thalamus" in the introduction and Figure 1 specifically denotes the pulvinar nucleus. Furthermore, as prior work highlights the tecto-thalamo-cortical pathway (SC→pulvinar→superficial V1 layers), whether the observed superficial V1 effects might originate from this pathway need to be at least discussed?
Re: Thank you for your constructive comments. We acknowledge that this is indeed an important point to clarify. According to previous studies (Shipp, 2003; Benarroch et al., 2015), there are two thalamic pathways from the SC to V1: one through the pulvinar and the other through the LGN. The pulvinar pathway terminates in both the superficial and middle cortical layers, whereas the LGN pathway primarily terminates in the middle layers of V1. We have revised the Introduction (lines 129–137) to clarify these points:*"* *if the saliency signals originate in the SC, they would modulate activity more strongly in the middle and superficial cortical layers through the tecto-thalamo-cortical pathway (Shipp, 2003, 2004, 2007). Specifically, this pathway may relay through the pulvinar, terminating in the middle and superficial cortical layers, or through the LGN, which primarily targets the middle layers of V1. Overall, the middle layers receive stronger thalamic input than the superficial layers (Shipp, 2003). Thus, although the SC was not recorded in the current study due to the limited field-of-view of VASO fMRI, the laminar profiles of saliency signals recorded in the cortical regions can still be used to test the hypothesis whether these signals originate from a subcortical source."*

If the saliency signals transmitted through the colliculus-pulvinar pathway, the laminar response profiles in IPS should closely resemble those observed in V1, as the superficial layers of IPS also receive pulvinar input. Given tthe distinct laminar profiles of saliency in V1 and IPS, it is unlikely that saliency is transmitted exclusively via the pulvinar pathway. We have included this point in the Discussion (lines 362–364): *"The saliency signal observed in the superficial layers of V1 is unlikely to originate from the colliculus–pulvinar pathway, as this would have resulted in a similar laminar profile in IPS (Figure 1)."*.

Reference:

Benarroch, E. E. (2015). Pulvinar: Associative role in cortical function and clinical correlations. *Neurology*, *84*(7), 738–747. https://doi.org/10.1212/wnl.0000000000001276

Shipp, S. (2003). The functional logic of cortico-pulvinar connections. *Philosophical Transactions of the Royal Society B: Biological Sciences*, *358*(1438), 1605–1624. https://doi.org/10.1098/rstb.2002.1213

Shipp, S. (2004). The brain circuitry of attention. *Trends in Cognitive Sciences*, *8*(5), 223–230. https://doi.org/10.1016/j.tics.2004.03.004

Shipp, S. (2007). Structure and function of the cerebral cortex. *Current Biology*, *17*(12), R443–R449. https://doi.org/10.1016/j.cub.2007.03.044

2 minor inconsistencies in data visualization warrant verification. For instance, in Figure S3 (IPS superficial layers) and Figure S6 (V1 deeper/middle layers), the distribution of individual data points appears partially misaligned with the plotted mean values. 
Re: Thank you for bringing these issues to our attention. We apologize for the errors in figure preparation. We have corrected these figures (reproduced below), and carefully checked all figures in both the main text and supplementary materials.

**Figure R2.** Revised figures in the manuscript.

3 while the VASO sequence effectively minimizes macrovascular contamination and improves spatial specificity, residual laminar signal-to-noise ratio (SNR) biases may persist. For example, Figure 3 illustrates consistently elevated superficial-layer signals across regions (green traces). Please rule out the possibility that superficial V1 effect is contaminated by the SNR differences across layers.
Re: Thank you for the constructive comments. We agree that controlling for potential confounds from laminar SNR differences is important for interpretation of the laminar fMRI results.

To explicitly address how SNR differences across layers influence the laminar pattern of the saliency signal, we calculated the laminar profile of temporal SNR (tSNR) and the contrast-to-noise ratio (CNR) in our regions of interest (Figure S10A). Then, saliency-sensitive responses were normalized using the slope of the tSNR or the CNR across layers (Figure S10B&C). After correcting the SNR differences across layers, the saliency signal in the superficial layers of V1 remained significantly stronger than in the middle and deep layers, while the IPS signal was strongest in the middle layers. These findings confirm that the observed laminar profile—especially the superficial V1 effect—was not attributable to SNR differences across layers.

We have added an explanation of the effects of laminar SNR differences on saliency-sensitive responses in the revised Results section (lines 272–275):*"To account for differences in signal-to-noise ratio (SNR) across cortical layers in the observed laminar profile, we normalized the saliency-sensitive responses* $S_{fMRI}$ *by the SNR at each cortical depth. The normalized responses still exhibited similar laminar profiles (Figure S10)."*

**Figure S10.** (A) The tSNR and CNR of CBV response across cortical depth in the foreground ROIs. tSNR: temporal signal-to-noise ratio, mean signal divided by the standard deviation. CNR: contrast-to-noise ratio, signal difference between stimulus and fixation periods divided by the standard deviation. (B) Saliency-sensitive responses normalized by tSNR. (C) Saliency-sensitive responses normalized by CNR. Error bars represent the standard error of the mean. *, ** and *** indicate p < 0.05, p < 0.01, p < 0.001, respectively.

4 the x-axis label in Figure 4A requires clarification. Based on Figure 2B, this axis does not appear to represent behavioral sensitivity SS(behavior). A precise definition of this metric is essential for reproducibility and data interpretation.
Re: We thank the reviewer for pointing out this unclear aspect and apologize for the typo in the mathematical definition of *behavioral sensitivity to saliency* ${SS}_{behavior}$. To enable a more direct and meaningful comparison with the normalized fMRI saliency signal, the behavioral saliency sensitivity ${SS}_{behavior}$ was also normalized by each participant’s mean behavioral sensitivity across different orientation contrasts (as in Figure 4A).

We have corrected the formula for ${SS}_{behavior}$ in the revised manuscript (lines 181–182). Data in Figure 4A were correct.

5 While fixation stability was compared across orientation contrast conditions, a more rigorous approach might involve analyzing gaze position distributions (e.g., heatmaps or dispersion metrics) across different stimulus positions.
Re: Thank you for the constructive comments. Unfortunately, we did not record the precise onset time of individual texture stimuli. We apologize for this limitation.

Since the foreground bars were presented in lower visual field, if participants’ gaze had been attracted by the foreground bars, we would expect to observe a higher number of fixation samples below rather than above the center of the fixation cross. To test this, we analyzed the distribution of gaze positions below the center of the fixation cross across the different orientation contrast conditions (Figure S3C). The results showed no significant difference among three orientation-contrast conditions, and the percentage of the fixation location below (rather than above) the cross is approximately 50%, suggesting that participants maintained good fixation when the foreground bars were presented in the lower visual field.

We have added this analysis and its results to the revised Results section (lines 344–348):*"The distributions of gaze positions below the center of the fixation cross also showed no significant difference among the three θ conditions (F_2,22_ = 2.197, p = 0.135, BF_10_ = 0.736, weak evidence for H_0_) (Figure S3C), suggesting that participants maintained good fixation when the foreground bars were presented in the lower visual field."*

**Figure S3.** (A) The group-averaged heat maps of gaze density in the entire session. (B) The bivariate contour ellipse area (BCEA) of fixation distribution showed no significant difference across θ conditions (F_2,22_ = 0.291, p = 0.750, BF_10_ = 0.227, moderate evidence for H_0_: no difference across θ conditions). Error bars represent the standard deviation of the means. (C) The percentages of the gaze positions (gaze density) below the center of the fixation point showed no significant difference across θ conditions (F_2,22_ = 2.197, p = 0.135, BF_10_ = 0.736, weak evidence for H_0_). The dash lines indicate the region of the gaze positions selected for analysis.

6 Please provide details on the number of voxels included in each laminar ROI across regions, as the activation in IPS in Figure 2D seems quite weak.
Re: Thank you for the comment. We have added detailed information (mean and SEM across participants) about the number of voxels included in each ROI in the ROI definition section of Methods (lines 599–602). Please note that, due to the spatial up-sampling (factor of 2) in our analysis, the voxel counts may be larger than those typically reported in conventional fMRI studies without up-sampling.

Reviewer #3: This manuscript reports the results from a psychophysical and high-resolution 7T fMRI study assaying the laminar profile of saliency modulations of neural activity evoked by task-irrelevant stimuli. The authors show evidence that superficial layers of V1 are more sensitive to changes in stimulus salience than middle/deep layers, consistent with a mechanism whereby feature contrast is computed locally in early visual cortex and transmitted to other regions, like parietal cortex. IPS was sensitive to changes in salience, but with a different laminar profile more consistent with receiving feedforward input. V1 superficial layer sensitivity to salience (difference in activation for high - low salience stimuli) correlated with individual participant sensitivity to differences in salience measured psychophysically outside the scanner (in a task where the salient location was reported, and thus task-relevant). Finally, the authors compare whether activation in V1 superficial layers drives activation in IPS middle layers, as predicted, using a gPPI analysis (version of functional connectivity), and show that connectivity is stronger when salience is greater, a result missing from feedback connections from deep IPS to deep V1 layers. The authors conclude that this establishes a key role of V1 in computing a saliency map, which is fed forward to later regions like IPS, consistent with the V1 salience hypothesis.

Overall, this is a strong study. The data is interesting, and the difference in relative activation profiles across layers between high- and low- salience conditions appears quite solid. I have some comments about the experiment design and reporting of results, as well as the degree to which conclusions from this study can be generalized to salience computations for other types of visual features. It will certainly be of interest to vision scientists, and likely to systems neuroscientists more broadly.

Major comments:
1.	Throughout the manuscript, the authors discuss 'salience maps', which per my understanding index the local feature contrast at specific locations in the visual environment, typically in retinotopic coordinates. But, the actual experiment doesn't seem to address spatial sensitivity whatsoever (as far as I can tell). This is because in the fMRI study, it seems that each block contains both trials containing salient locations on the left and right side of the screen (randomly chosen for each of the 60 trials per 30 s block). This means both hemispheres' foreground-selective ROI should be stimulated, which is I suppose some degree of evidence for a 'map' (at least in V1), but this also means it's not possible to establish spatial localization of salience computations on each block. If instead left-only and right-only blocks were included, the authors could verify that results hold when the salient stimulus is presented in the contralateral visual field for each ROI (rather than anywhere on the screen), and this would allow for better testing of the functional connectivity, because the specific prediction seems to be that information about the salience of a visual field location is transmitted from V1 to IPS between corresponding parts of their respective retinotopic maps. I understand that acquiring more data is infeasible, but this limitation should be clearly considered, and in my view the impact of the limitation on the conclusions may be somewhat broad. (I'll note that it's very obvious that the signal in V1/V2 is exceedingly likely to be driven by the foreground stimulus in the contralateral visual field due to small pRFs and clear retinotopic organization. But the same isn't as clearly true for IPS, due both to large pRF size and less clear retinotopic organization. Because the results rely on this comparison, I think this issue needs to be discussed).

Re: Thank you for your insightful comments. We agree that IPS has larger pRFs and has a less clear retinotopic organization compared to those in early visual areas. However, we would like to clarify that the retinotopic ROIs in IPS were accurately defined using independent retinotopic localizers, in which left and right stimuli were presented in separate blocks using the same size and at the same location as those for the foreground regions in the main experiment. Figure S13A shows the activation map to the localizer stimuli in a representative participant. IPS activations were strongly lateralized: stimuli presented to the left visual field activated the right IPS, while those presented to the right visual field activated the left IPS. This lateralization is consistent with prior findings of clear retinotopic organization in posterior IPS (Swisher et al., 2007), where our ROIs are primarily located (Figure S13B). Therefore, although some degree of ipsilateral activation in IPS cannot be ruled out due to its large pRFs, the ROI-averaged responses are still expected to be predominantly driven by contralateral stimuli, especially in the posterior IPS. Furthermore, to ensure retinotopic correspondence between V1 and IPS activity, the gPPI analysis of effective connectivity was conducted separately within each hemisphere, and the results were subsequently averaged across hemispheres. Importantly, our conclusion depends on the laminar pattern rather than the retinotopic specificity of saliency signals in IPS. Saliency signals to ipsilateral stimuli due to coarse retinotopic map in IPS is unlikely to have a different laminar pattern as those to contralateral stimuli.

In the main experiment, salient singletons appeared in the lower left or right visual field in random order and at random intervals within each block. This design was chosen to minimize top-down anticipatory effects on the brain responses to our singleton stimuli. We agree that separating responses to the contralateral singletons would better support analyses of spatial specificity and inter-regional connectivity. However, achieving this would require an event-related design, which is not feasible with the current VASO sequence due to its relatively low temporal resolution. Furthermore, doing this would risk adding top-down contributions to our IPS activations since observers could anticipate the location of the foreground.

We have now revised the manuscript to discuss these issues in the Discussion (lines 409–421): *"Considering the slow acquisition speed of the VASO sequence, and to minimize top-down anticipatory effects, we employed a block design in which a salient foreground region was presented in the lower-left or lower-right visual field in a random order and at random intervals within a 30-s block. In principle, with such a design, we cannot exclude the possibility that the responses to ipsilateral foreground stimuli were also in the ROI-averaged response of IPS. However, our IPS ROIs were primarily located in the posterior IPS (Figure S13), which exhibits strong lateralization and clear retinotopic organization (Swisher et al., 2007). Therefore, the saliency-sensitive responses in the IPS should be predominantly driven by the contralateral foreground. Importantly, our conclusion depends on the laminar pattern rather than the retinotopic specificity of saliency signals in the IPS. Nevertheless, an event-related design would allow for measuring fMRI responses to individual foreground events. Achieving this will require further development of high-resolution fMRI methods with faster acquisition speed**."*.

**Figure S13.** (A) Localizer activations to the left- or right-side stimuli in the IPS of a representative participant. (B) Group-averaged ROI volume in IPS subregions. Error bars represent SEM across participants. Each gray dot represents data from one participant.

Reference:

Swisher, J. D., Halko, M. A., Merabet, L. B., McMains, S. A., & Somers, D. C. (2007). Visual Topography of Human Intraparietal Sulcus. *The Journal of Neuroscience*, *27*(20), 5326–5337. https://doi.org/10.1523/jneurosci.0991-07.2007

2.	In this study, the salient stimuli are never task-relevant (inside the scanner). This is enforced using a fixation task. However, the difficulty of the fixation task is not mentioned. What was participant performance on the fixation task? I worry that if the task is too easy, the participants can be simultaneously monitoring the peripheral stimuli, and so aspects of the results could reflect covert selection/attention.

Re: Thank you for raising this important point. In the saliency fMRI experiment, participants were instructed to count the number of subtle luminance reductions at fixation (approximately 25% dimming), while ignoring the peripheral texture stimuli. This required sustained attention to the fixation point to perform the task accurately. The number of luminance reductions varied across runs and was reported after each run, with feedback provided afterward.

The fixation task was designed to be attentionally demanding by the subtlety and the large number of the luminance changes in each run. Consequently, the reported number of luminance changes was correct in only 32.0% of the runs on average, and deviated from the correct number by 4.2% on average (Figure S2). Hence, the task was far from trivial, yet the participants remained engaged and performed well. Furthermore, eye-tracking data confirmed that fixation was stable and not systematically biased toward the foreground stimuli (Figure S3).

Taken together, these results suggest that the fixation task was sufficiently difficult and the covert attention to the peripheral stimuli should be minimal.

Our revised manuscript reports the task performance in Results (lines 222–223): *"The performance indicates that the task was sufficiently challenging and yet participants remained engaged and performed well (Figure S2).* "

3.	Additionally, related to task relevance: it may be useful to consider how computations in salience maps like that measured here are expected to change across manipulations of task relevance of salient stimuli. Clearly, the task-irrelevant stimuli can evoke location-selective fMRI responses - but what if there were a competing task, like search for color singleton?

Re: Thank you for this insightful comment regarding the influence of task relevance on saliency-related fMRI responses. The current study was designed to isolate bottom-up saliency, which refers to attentional capture driven by goal-irrelevant, exogenous visual inputs. To minimize top-down influences, the orientation singletons were intentionally rendered task-irrelevant, at a peripheral location (away from the task-relevant central fixation), with unpredictable time and hemifield of their appearance.

To isolate neural responses driven by bottom-up saliency from those influenced by task goals, the peripheral texture stimuli containing the orientation singletons were made task-irrelevant by assigning participants a demanding central fixation task. This task required sustained, space-based top-down attention at the fixation point, thereby directing attention away from the salient peripheral stimuli. In contrast, if participants were instead asked to perform a task such as searching for a color singleton, the locus of top-down, goal-directed attention would vary across trials depending on the target’s position. This would introduce potential confounds in the neural responses, particularly when the task-relevant color singleton appears near the task-irrelevant orientation singleton. Such confounds are likely to be stronger in higher-level visual areas, which integrate both bottom-up and top-down signals—as shown in monkey LIP, where neurons encode both saliency and task goals (Bisley & Goldberg, 2010). These confounds would increase the variability in ROI responses, making it more difficult to interpret saliency-specific effects. Thus, the use of a central fixation task—where top-down attention remains fixed and spatially separated from the salient stimuli—is better suited for isolating bottom-up saliency responses in our study.

We have now clarified our experimental design in Introduction (lines 142–146):*"To link the fMRI responses with the bottom-up saliency of the foreground region rather than top-down task goals, the fMRI responses were collected from participants who were performing a demanding fixation task at a central fixation cross away from the foreground region. Consequently, the peripheral texture stimuli, including the foreground region, were task-irrelevant."*

Reference:

Bisley, J. W., & Goldberg, M. E. (2010). Attention, Intention, and Priority in the Parietal Lobe. *Annual Review of Neuroscience*, *33*(1), 1–21. https://doi.org/10.1146/annurev-neuro-060909-152823

4.	The authors interpret their findings - which I'm overall convinced by - as evidence that V1 acts as a salience map. Is it valid to interpret this as a salience map given only a single feature - for which V1 is tuned - is tested? Would the authors predict that other salient features, like color, shape, or motion direction (e.g., Burrows & Moore, 2009), are encoded in a similar manner, with V1/IPS responses scaling as a function of feature contrast and correlating with behavior? Or would these types of computations occur in other regions? Some other recent fMRI work (e.g., Thayer & Sprague, 2023; Bogler et al, 2011; Poltoratski et al, 2017) may speak to this, and is not cited in the present manuscript.

Re: Thank you for the important and thoughtful comment regarding the generalizability of our findings beyond orientation saliency. While our study focused on orientation saliency—due to V1’s well-known tuning to orientation—numerous behavioral studies have supported the idea that V1 also contributes to saliency computations based on other low-level features, including color, motion direction, eye of origin, and their combinations (Koene & Zhaoping, 2007; Zhaoping, 2008; Zhaoping & May, 2007; Zhaoping et al 2009, Zhaoping & Zhe, 2015). These findings suggest that V1 computes a salience map based on simple feature contrasts that fall within its tuning properties.

Saliency-related signals have been observed in higher order visual areas (Thayer & Sprague, 2023; Bogler et al., 2011; Poltoratski et al., 2017), although it is unclear whether these signals are inherited from V1 or computed within these regions. However, it is important to emphasize that saliency, defined as exogenously guided attentional attraction, guides bottom-up attentional selection in a rapid and transient manner (Donk & Van Zoest, 2008; Müller & Rabbitt, 1989; Nakayama & Mackeben, 1989). V1 saliency signals occur earlier in the visual hierarchy and are more closely linked with the transient nature of bottom-up attentional shifts. Furthermore, when we restrict to attentional guidance by very salient stimuli (so that reaction times of the attentional shifts are short) due to feature contrast in color, orientation, motion direction, and eye of origin, behavioral data (such as Koene & Zhaoping 2007, Zhaoping 2008, Zhaoping et al 2009, Zhaoping & Zhe 2015) suggest that V1 properties alone can fully account for the data. In other words, including contributions from cortical areas beyond V1 to saliency contribution would lead to disagreement with the behavioral data. Future studies are highly desirable to find out whether this conclusion holds in other saliency behavior.

We have discussed these points in the revised manuscript (lines 422–436):*"Although this study only investigated the neural mechanisms of saliency based on orientation contrast, previous behavioral studies suggest that V1 also computes saliency from other low-level features such as color, motion direction, eye of origin, and their combinations (Koene & Zhaoping, 2007; Zhaoping, 2008; Zhaoping & May, 2007; Zhaoping et al 2009, Zhaoping & Zhe, 2015). Thus, our findings are likely to generalize to saliency due to contrast in all the basic feature dimensions. Although saliency-related signals have also been observed in higher brain regions (Thayer & Sprague, 2023; Bogler et al, 2011; Poltoratski et al, 2017), it remains unclear whether these signals are inherited from V1 or computed within these brain regions themselves. Meanwhile, saliency signals computed by V1 should be the fastest for rapid deployment of bottom-up attentional shifts. To make the deployment rapid, the direct anatomical projection from V1 to SC should be favored compared to indirect projections from V1 to SC via higher cortical areas such as the parietal and frontal cortices. Meanwhile, when bottom-up saliency is combined with top-down factors to guide attention (Bisley & Goldberg, 2010), in cooperation or in competition, the feedforward connectivity such as the ones investigated in this study, should play important roles.".*

Reference:

Bisley, J. W., & Goldberg, M. E. (2010). Attention, Intention, and Priority in the Parietal Lobe. *Annual Review of Neuroscience*, *33*(1), 1–21. https://doi.org/10.1146/annurev-neuro-060909-152823

Bogler, C., Bode, S., & Haynes, J.-D. (2011). Decoding Successive Computational Stages of Saliency Processing. *Current Biology*, *21*(19), 1667–1671. https://doi.org/10.1016/j.cub.2011.08.039

Donk, M., & Van Zoest, W. (2008). Effects of Salience Are Short-Lived. *Psychological Science*, *19*(7), 733–739. https://doi.org/10.1111/j.1467-9280.2008.02149.x

Koene, A. R., & Zhaoping, L. (2007). Feature-specific interactions in salience from combined feature contrasts: Evidence for a bottom–up saliency map in V1. *Journal of Vision*, *7*(7), 6. https://doi.org/10.1167/7.7.6

Li, Z. (2008). Attention capture by eye of origin singletons even without awareness—A hallmark of a bottom-up saliency map in the primary visual cortex. *Journal of Vision*, *8*(5), 1. https://doi.org/10.1167/8.5.1

Müller, H. J., & Rabbitt, P. M. (1989). Reflexive and voluntary orienting of visual attention: Time course of activation and resistance to interruption. *Journal of Experimental Psychology: Human Perception and Performance*, *15*(2), 315–330. https://doi.org/10.1037/0096-1523.15.2.315

Nakayama, K., & Mackeben, M. (1989). Sustained and transient components of focal visual attention. *Vision Research*, *29*(11), 1631–1647. https://doi.org/10.1016/0042-6989(89)90144-2

Poltoratski, S., Ling, S., McCormack, D., & Tong, F. (2017). Characterizing the effects of feature salience and top-down attention in the early visual system. *Journal of Neurophysiology*, *118*(1), 564–573. https://doi.org/10.1152/jn.00924.2016

Thayer, D. D., & Sprague, T. C. (2023). Feature-Specific Salience Maps in Human Cortex. *The Journal of Neuroscience*, *43*(50), 8785–8800. https://doi.org/10.1523/JNEUROSCI.1104-23.2023

Zhaoping, L., Guyader, N., & Lewis, A. (2009). Relative contributions of 2D and 3D cues in a texture segmentation task, implications for the roles of striate and extrastriate cortex in attentional selection. *Journal of Vision*, *9*(11), 20–20. https://doi.org/10.1167/9.11.20

Zhaoping, L., & May, K. A. (2007). Psychophysical Tests of the Hypothesis of a Bottom-Up Saliency Map in Primary Visual Cortex. *PLoS Computational Biology*, *3*(4), e62. https://doi.org/10.1371/journal.pcbi.0030062

Zhaoping, L., & Zhe, L. (2015). Primary Visual Cortex as a Saliency Map: A Parameter-Free Prediction and Its Test by Behavioral Data. *PLOS Computational Biology*, *11*(10), e1004375. https://doi.org/10.1371/journal.pcbi.1004375

5.	Statistics: at present, a core part of the central conclusion rests on the results shown in Fig. 3A - the depth profile of the saliency signal changes across ROIs. However, the critical interaction between depth and ROI is not tested (that I could see).

Re: We would like to thank the reviewer for the constructive comment. In the revised version, we have conducted a two-way repeated-measures ANOVA with cortical depths (deep, middle, superficial) and ROIs (V1, V2, IPS) as within-subject factors to compare the depth profile of saliency-sensitive fMRI responses ${SS}_{fMRI}$. This ANOVA revealed a significant interaction effect between depth and ROI (F_4,76_ = 6.052, p = 0.002), indicating that the effect of cortical depth on the saliency signal depends on the ROI.

We have added this result and the corresponding statistics in Results (lines 262–264): *"* *A significant interaction effect was found between depth and ROI, indicating that the effect of the cortical depth on the* ${SS}_{fMRI}$ *depended on the ROIs (F_4,76_ = 6.052, p = 0.002).* "

Minor comments:
1.	Neural/behavioral correlation - right now, the 'winning' layer is used for each region, and the authors conclude a selective correlation between V1 superficial layer salience modulation and behavioral salience sensitivity. But is this result selective to superficial layers in V1, rather than middle/deep layers of V1? All layers, ROIs should be tested for completeness.

Re: Thank you for this important suggestion. In response, we have now included the correlations between saliency-sensitive fMRI responses and behavioral sensitivity to saliency across all cortical depths in each ROI (see Figure S11). The results show that only the superficial layers of V1 exhibited a significant correlation with behavioral sensitivity to saliency.

**Figure S11.** Pearson’s correlation between ${SS}_{behavior}$ and ${SS}_{fMRI}$ across three cortical depths in V1, V2, and IPS. Each circle represents one participant.

2.	In the connectivity analyses, only gPPI differences between 90 and 15 deg conditions are reported. Is the gPPI positive for each of the values used to compute the difference, for each layer/ROI tested? If both values are negative, but gPPI is less negative for one condition than another, this would be more challenging to interpret as support for the authors' hypothesis

Re: Thank you for raising this important point. Following your suggestion, we now present the gPPI beta values for both the 90° and 15° conditions in Figure S12A. The results show that the gPPI terms were positive for both conditions in the feedforward and feedback connections, while three of them were significant from zero (feedforward 90°: t_19_ =5.853, p < 0.001; feedforward 15°: t_19_ = 1.253, p = 0.225; feedback 90°: t_19_ = 4.350, p < 0.001; feedback 15°: t_19_ = 3.734, p = 0.001). Moreover, a significant difference between the two orientation-contrast conditions was observed only for the feedforward connection, consistent with our hypothesis (feedforward 90° vs. 15°: t_19_ = 4.384, p < 0.001; feedback 90° vs. 15°: t_19_ = 2.041, p = 0.055). The interaction between pathways and conditions was also significant (F_1,19_ = 10.067, p = 0.005). It is worth to mention that, to facilitate the comparison of gPPI beta values between feedforward and feedback connections, we normalized the ROI timeseries by Z-score in this revision. We have updated the gPPI results in the main text (lines 325–326).

**Figure S12.** (A) Beta values of gPPI terms for the 90° and 15° conditions. Each gray dot represents one participant. Error bars represent the standard errors of the mean across participants. One sample t-tests indicate significant positive connectivity in 90° of the feedforward pathways, 90° and 15° of the feedback pathways (feedforward 90°: t_19_ =5.853, p < 0.001; feedforward 15°: t_19_ = 1.253, p = 0.225; feedback 90°: t_19_ = 4.350, p < 0.001; feedback 15°: t_19_ = 3.734, p = 0.001). Paired t-tests indicate significant connectivity difference only in the feedforward pathway (feedforward 90° vs. 15°: t_19_ = 4.384, p < 0.001; feedback 90° vs. 15°: t_19_ = 2.041, p = 0.055). A two-way rm ANOVA shows a significant interaction between pathways (feedforward and feedback) and θ conditions (90° and 15°) (F_1,19_ = 10.067, p = 0.005).

3.	In the gPPI analysis, the authors report a significant connectivity difference for feedforward connections, and no significant connectivity difference for feedback - but is there a difference between these differences?

Re: Thank you for your constructive comment. There was indeed a significant difference between the gPPI terms in feedforward and feedback connections (t_19_ = 3.173, p = 0.005), supporting that the saliency is more likely to transmit via the feedforward pathway. We have updated the statistical results in the main text (lines 326-328):*“* *A paired t-test showed that the feedforward connectivity difference* $\beta_{FF}\left( 90^{\circ} \right)-\beta_{FF}(15^{\circ})$ *was significantly stronger than the feedback connectivity difference* $\beta_{FB}\left( 90^{\circ} \right)-\beta_{FB}(15^{\circ})$ *(t_19_ = 3.173, p = 0.005).”*

4.	In Fig. 2D, it looks like there are 3 disjoint clusters identified by the localizer in IPS. Were all significant voxels included? These may aggregate across different retinotopic IPS regions (discussed in detail in the Wang et al 2015 paper describing the atlas used by the authors). Is this the case for all hemispheres/subjects?

Re: Thank you for bringing the important point to us. In the ROI definition of IPS, we selected significant clusters (voxel p = 0.05, cluster size >= 120 upsampled voxels) activated by the localizer stimuli within the anatomical mask of IPS on the cortical surface.

In the added new Figure S13A, we present localizer activations corresponding to the retinotopic location of the foreground bars in IPS in a representative participant (Borders of IPS subregions marked with dash lines). To clarify how the analyzed ROI distribute across different retinotopic IPS regions in all subjects, we examined the distribution of these clusters among IPS subregions (Figure S13B).

Most hemispheres contained 2 or 3 significant clusters spanning multiple IPS subregions, with many clusters located in the posterior IPS. Although most participants exhibited significant localizer activation in the IPS (as shown in Figure S13A), three participants (five hemispheres) did not show significant clusters at the cluster defining threshold. In these cases, we slightly relaxed the cluster defining threshold until two clusters could be identified for each hemisphere. On average, each hemisphere contained 2.625 ± 0.807 clusters.

We added the corresponding description in revised Method section (lines 591-595):*"* *Three participants (five hemispheres) did not show significant clusters at the threshold. In these cases, we slightly relaxed the cluster defining threshold until two clusters could be identified for each hemisphere. The foreground ROIs in IPS consisted of 2-3 clusters per hemisphere (2.625 ± 0.807 on average), mainly located in the posterior IPS (Figure S13B)."*

5.	The Methods mention that the pRF-based image space visualizations shown in Fig. 3C are 'linearly transformed' to align across participants (line 527-28). Please elaborate on this process, especially because there are not individual-subject pRF maps available. (additionally, perhaps the localizer stimulus could be useful here?)

Re: As you noted, individual pRF maps were not directly measured in this study. Instead, we used the localizer activation to adjust the group-level pRF parameters (from the Benson atlas) in a subject-specific manner. Specifically, for each hemisphere in one participant, we identified the cortical vertex that showed the maximal activation in the retinotopic localizer. We then extracted its corresponding polar angle (A_m_) and eccentricity (E_m_) from the pRF atlas.

The foreground bars located on screen at 127.88° in polar angle and 4.28° in eccentricity (assuming perfect fixation). To align the subject's pRF representation with this reference location, we linearly transformed the pRF parameters of all vertices as follows:

Corrected angle:  $A_{c}= \frac{A}{A_{m}} \times127.88^{\circ}$

Corrected eccentricity:  $E_{c}= \frac{E}{E_{m}} \times4.28^{\circ}$

Here, A and E denote the original pRF parameters from the Benson atlas, and A_c_ and E_c_ are the corrected values.

No additional spatial transformations were applied beyond this localizer-based linear scaling. The final visual field map was reconstructed by weighting each node’s CBV response by its corrected pRF estimate.

We have now added this explanation to the Methods section (lines 609–615):*"Specifically, angle and eccentricity parameters (*$A_{m}$ *and* $E_{m}$*) were obtained for the cortical vertex showing the maximum localizer activation, separately for each hemisphere. The corrected pRF parameters were defined as:* $A_{c}= \frac{A}{A_{m}} \times127.88^{\circ}$*,* $E_{c}= \frac{E}{E_{m}} \times4.28^{\circ}$*. Here, the polar angle of 127.88° and eccentricity of 4.28° correspond to the screen location of foreground bars.* $A$ *and* $E$ *are the original pRF parameters provided by the Benson atlas (Benson et al., 2018)."*

References:

Benson, N. C., Jamison, K. W., Arcaro, M. J., Vu, A. T., Glasser, M. F., Coalson, T. S., Van Essen, D. C., Yacoub, E., Ugurbil, K., Winawer, J., & Kay, K. (2018). The Human Connectome Project 7 Tesla retinotopy dataset: Description and population receptive field analysis. *Journal of Vision*, 18(13), 23. https://doi.org/10.1167/18.13.23

6.	What were the contrast steps used for the staircases in the psychophysics experiment?

Re: In the psychophysical experiment, bar luminance was adjusted by a 3-down-1-up staircase procedure to determine the participant’s contrast sensitivity for detecting the foreground. The initial Michelson contrast was 0.2863, decreased by 15% (0.2863/1.15) if participant made three correct responses in a row, or increased by 15% (0.2863*1.15) following each incorrect response. We have added this information to the Methods section (line 483–484):*"The initial contrast of the staircase procedure was 0.2863 and changed by 15% in each step.".*

7.	Fig. 3B shows the effect of salience on fMRI activation in volume space for an example participant. However, it appears that this activation is masked by the localizer-defined ROI. It may be helpful to illustrate unmasked activation profiles, including, if possible, unmasking CSF/GM to show the spatial specificity of signals attained

Re: We would like to thank the reviewer for the helpful suggestions. We have now included a laminar profile of CBV response with CSF and WM data in Figure S6.

**Figure S6.** Laminar profile of CBV responses extended to into WM and CSF. Top panel: CBV responses ($S_{fMRI}$ in percent signal change) to the orientation foregrounds in V1, V2, and IPS. Bottom panel: CBV response difference between the 90° and 15° foreground conditions (${SS}_{fMRI}= S_{fMRI}\left( {90}^{o} \right)- S_{fMRI}\left( {15}^{o} \right)$). Error bars represent SEM. WM: white matter, GM: gray matter, CSF: cerebrospinal fluid.

8.	More details should be provided about how activation was mapped from voxels to layers (i.e., the demixing procedure/associated procedures should be specified in the Methods if possible)
We thank the reviewer for highlighting the need to clarify the demixing procedure. Briefly, two equi-volume intermediate surfaces between the white matter and pial surfaces were generated through surface segmentation. Voxel-wise layer weights were then calculated using the mripy toolbox (https://github.com/herrlich10/mripy), resulting in a matrix representing the proportion of each voxel’s volume assigned to five compartments: white matter (WM), cerebrospinal fluid (CSF), and three gray matter layers (superficial, middle, and deep).

To extract layer-specific responses, we applied a spatial regression using a general linear model (GLM):

Y = X × B + U

Here, Y is an n × 1 vector of voxel responses within the ROI (n = number of voxels), X is the n × 5 matrix of voxel-wise compartment weights, B is the 5 × 1 vector of estimated responses for each layer, and U is the residual error. This regression effectively unmixes the laminar signals, minimizing cross-layer contamination. This approach is consistent with prior studies (e.g., Kok et al., 2016; Liu et al., 2021).

We have now added a detailed description to the Methods section (lines 576–583):*"Specifically, We applied a general linear model (GLM) to estimate the response associated with each cortical layer: Y = X × B + U, where Y is an n × 1 vector containing voxel responses within the ROI (n denotes the total number of voxels), X is an n × 5 layer weight matrix representing the proportion of each voxel’s volume assigned to the five compartments (three cortical layers, white matter, and CSF), and B is a 5 × 1 vector of the estimated layer-specific responses. The residual term U is an n × 1 vector capturing deviations from the least-squares fit. This unmixing approach helps to minimize correlations in activity across different cortical layers."*

References:

Kok, P., Bains, L. J., van Mourik, T., Norris, D. G., & de Lange, F. P. (2016). Selective Activation of the Deep Layers of the Human Primary Visual Cortex by Top-Down Feedback. *Current Biology*, *26*(3), 371–376. https://doi.org/10.1016/j.cub.2015.12.038

Liu, C., Guo, F., Qian, C., Zhang, Z., Sun, K., Wang, D. J., He, S., & Zhang, P. (2021). Layer-dependent multiplicative effects of spatial attention on contrast responses in human early visual cortex. *Progress in Neurobiology*, *207*, 101897. https://doi.org/10.1016/j.pneurobio.2020.101897
