# Supplementary material for: The superficial layers of the primary visual cortex create a saliency map that feeds forward to the parietal cortex
Source: PLoS Biol. 2025 Oct 14;23(10):e3003159. doi: 10.1371/journal.pbio.3003159 (PMC12543185; doi:10.1371/journal.pbio.3003159)
Supplement: Attachment — Submitted filename: Response_to_Reviewers_auresp_3.docx [file pbio.3003159.s018.docx]

Reviewer #3: I appreciate the authors' very careful attention to my previous comments, and to comments raised by the other reviewers. In my view, the manuscript is much stronger after these edits. My only remaining comment concerns the scaling procedure used to adjust atlas-based pRF values to align with a localizer. I think this is overall a clever and efficient solution to the problem of estimating a rough mapping between single-subject activation patterns and visual field maps in the absence of subject-specific retinotopy, but I'm a bit worried about the selection of scaling procedure implemented (see below)
1.	Scaling: In their detailed reply to my previously-raised point 5, the authors helpfully describe how they adjusted the pRF parameters pulled from the Benson atlas based on the participant's localizer data. However, I'm a bit confused/concerned about the scaling operation applied to polar angle - why scale linearly in polar coordinates, rather than linearly in Cartesian coordinates? If I'm understanding correctly, this type of polar angle scaling is similar to opening/closing a handheld fan - voxel pRF positions would rotationally expand/contract from 0 deg. Such a scaling approach does not account for the circular nature of the variable (i.e., a >1 scaling term will mean polar angles of 359 will 'loop back around'; a <1 scaling term would leave a portion of the visual field empty). Additionally, it seems that this approach will necessarily result in some voxels 'moving' across the horizontal hemifield boundary (e.g., a voxel with an original polar angle of 195 deg, assuming 180 deg is lower vertical meridian, could be scaled below 180 deg, which would move the pRF across the meridian). A linear scaling in Cartesian coordinates would be more appropriate. Alternatively, if the scaling factors are demonstrated to be quite small (~10% or less for polar angle adjustments), I'm not as concerned, as the impact of this choice would be minimal.

Re: We thank the reviewer for the careful consideration and constructive feedback on our methods. In our analysis, we reconstructed the visual field maps separately for the left and right hemispheres, which constrained the polar angle before correction within 0º~180º (or -180º~0º). This would not allow voxels with an original polar angle greater than 180 deg to move across the vertical meridian. However, within each hemisphere, the original scaling method could still cause the pRF of a voxel to move across (a>1) or away (a<1) from the vertical meridian, leading to an enlarged or an empty space in the reconstructed visual field map (a<1 was illustrated below in Figure R1).

Figure R1. Illustration of missing visual field (a<1) with the scaling method in one hemisphere. The red sphere indicates the activation center of the localizer, and the dashed circle marks the true position of the foreground region. The blue region represents the reconstructed visual field from the right hemisphere. Left: visual field map reconstructed using the original pRF parameters. Right: visual field map after correction, where part of the visual field is lost.

To address this issue, we optimized the correction method as follows:

$$A_{c}= \left\{ \begin{aligned} \frac{A}{A_{m}} \times127.88^{\circ}, 0^{\circ}\leq A\leq A_{m} \\ 180^{\circ} - \frac{180^{\circ}-A}{180^{\circ}- A_{m}} \times\left( 180^{\circ} -127.88^{\circ} \right), A_{m}<A\leq180^{\circ} \end{aligned} \right.$$

This new scaling method avoids the cross over and empty space in the reconstruct visual field.

Correction in Cartesian coordinates is also a feasible approach for handling errors in the original pRF parameters. However, simple linear scaling within each hemisphere could still cause empty spaces or crossover artefacts in the reconstructed visual field (Figure R1). Therefore, non-linear transformations would be required for a complete solution.

Finally, the scaling factors of polar angle ($|\frac{A_{c}}{A}-1|$) were indeed less than ~10% on average: 0.096 ± 0.086 (Mean ± STD across hemispheres). Thus, the correction is generally moderate.

We have updated the correction formula in the revised manuscript (line 613) and the corresponding visual field map in Figure 3C.

Figure 3C. Left: Surface activation maps of saliency-sensitive response (${SS}_{fMRI}$ in percent signal change) in different cortical depths of V1 in a representative participant (same participant as in B). Dashed circles indicate the location of foreground on the cortical surface. Right: Saliency maps (averaged across all participants) in image space.
